# Effects of long-acting, broad spectra anthelmintic treatments on the rumen microbial community compositions of grazing sheep

**DOI:** 10.1038/s41598-021-82815-y

**Published:** 2021-02-15

**Authors:** Christina D. Moon, Luis Carvalho, Michelle R. Kirk, Alan F. McCulloch, Sandra Kittelmann, Wayne Young, Peter H. Janssen, Dave M. Leathwick

**Affiliations:** 1 Grasslands Research Centre, AgResearch Limited, Palmerston North, New Zealand; 2 Invermay Research Centre, AgResearch Limited, Mosgiel, New Zealand; 3 Wilmar International Limited, WIL@NUS Corporate Laboratory, Centre for Translational Medicine, National University of Singapore, Singapore, Singapore

**Keywords:** Microbial communities, Animal physiology

## Abstract

Anthelmintic treatment of adult ewes is widely practiced to remove parasite burdens in the expectation of increased ruminant productivity. However, the broad activity spectra of many anthelmintic compounds raises the possibility of impacts on the rumen microbiota. To investigate this, 300 grazing ewes were allocated to treatment groups that included a 100-day controlled release capsule (CRC) containing albendazole and abamectin, a long-acting moxidectin injection (LAI), and a non-treated control group (CON). Rumen bacterial, archaeal and protozoal communities at day 0 were analysed to identify 36 sheep per treatment with similar starting compositions. Microbiota profiles, including those for the rumen fungi, were then generated for the selected sheep at days 0, 35 and 77. The CRC treatment significantly impacted the archaeal community, and was associated with increased relative abundances of *Methanobrevibacter ruminantium*, *Methanosphaera* sp. ISO3-F5, and *Methanomassiliicoccaceae* Group 12 sp. ISO4-H5 compared to the control group*.* In contrast, the LAI treatment increased the relative abundances of members of the *Veillonellaceae* and resulted in minor changes to the bacterial and fungal communities by day 77. Overall, the anthelmintic treatments resulted in few, but highly significant, changes to the rumen microbiota composition.

## Introduction

Anthelmintic treatment of adult ewes is practiced by about 80% of sheep farmers in New Zealand^1^, and many of these treatments use active compounds with broad-spectrum persistent activity. Treatment is undertaken with the expectation that parasitic helminths have a detrimental effect on ewe performance, and on that of their lambs, and that removing them will result in both the ewes and their lambs being heavier at weaning and that the ewes will be in better body condition. However, recent studies indicate that the effects of anthelmintic treatment of ewes are likely to be highly variable and will not always be positive^2–4^. Furthermore, on some farms more than half of the measured response to treatment was attributable to the trace elements that the products deliver, and not to the effect of anthelmintics removing the parasite burden^4^. It is clear that the interaction between parasites, the sheep and the administered anthelmintics are not as straight-forward as many farmers believe.

The microbiota of the rumen, the fermentative forestomach of ruminant animals, are essential for feed digestion and thus have a major influence on nutrition of the host animal. Interactions between parasites, anthelmintics and the rumen microbiota are poorly understood. A link between anthelmintic use and changes in the microbiota is not unreasonable, as the active ingredients in most long-acting anthelmintic products come from two classes of compounds, the benzimidazoles and macrocyclic lactones, both of which have activity against organisms other than nematodes. The benzimidazoles include albendazole, and are recognized as having antibacterial and antifungal activities^5^. Furthermore, the chemically-related compounds benomyl and carbendazim are commercially available as agricultural fungicides, and albendazole is available in several fungicide products. The macrocyclic lactones are characterised by the presence of a macrocyclic ring structure, and include the avermectins and milbemycins such as abamectin and moxidectin. Some compounds within this class are registered as antibiotics and anti-cancer drugs. Avermectins were originally reported to lack significant antibacterial or antifungal activity^6^, but have since been shown to inhibit growth of fungi^7^ and *Mycobacterium tuberculosis*^8^. To date, few studies have examined the impact of benzimidazoles and macrocyclic lactones on the animal gut microbiota, where the bacterial members of the communities have mainly been examined, and these studies have generally reported only minor alterations to the microbiota^9–11^. For example, no changes to the alpha or beta diversity, or the relative abundances of bacterial species were detected in healthy adult beagles as a result of using commercial anthelmintic treatments of febantel, pyrantel and praziquantel or fenbendazole^10^. Assessment of fenbendazole and moxidectin treatments used in quarantine practices showed that the effects of acclimation to the quarantine conditions on the gut microbiota of laboratory mice were greater than those of the anthelmintic treatments tested^11^. However, moderate changes to the abomasal, colon and faecal microbiota of moxidectin-treated *Haemonchus contortus*-infected goats were observed, which appeared to correlate strongly with the extent of parasite clearance^12^. In humans, albendazole was shown to have little impact on the gut bacterial communities of helminth-free subjects, but in parasite-infected subjects, interactions between treatments and infection level on the microbiota were apparent^13^.

The antimicrobial characteristics of anthelmintic compounds raise the possibility that continuous release of these actives into the rumen could have effects on the composition and activities of the rumen microbiota, potentially influencing animal performance. In this study we examined the impact of two commercially available long-acting anthelmintic products on the rumen microbiota of sheep, as our first step to investigate these interactions and understand the observed variability in response associated with anthelmintic treatments.

## Results

### Diversity of sheep rumen microbiota prior to treatment administration

**Figure 1:**
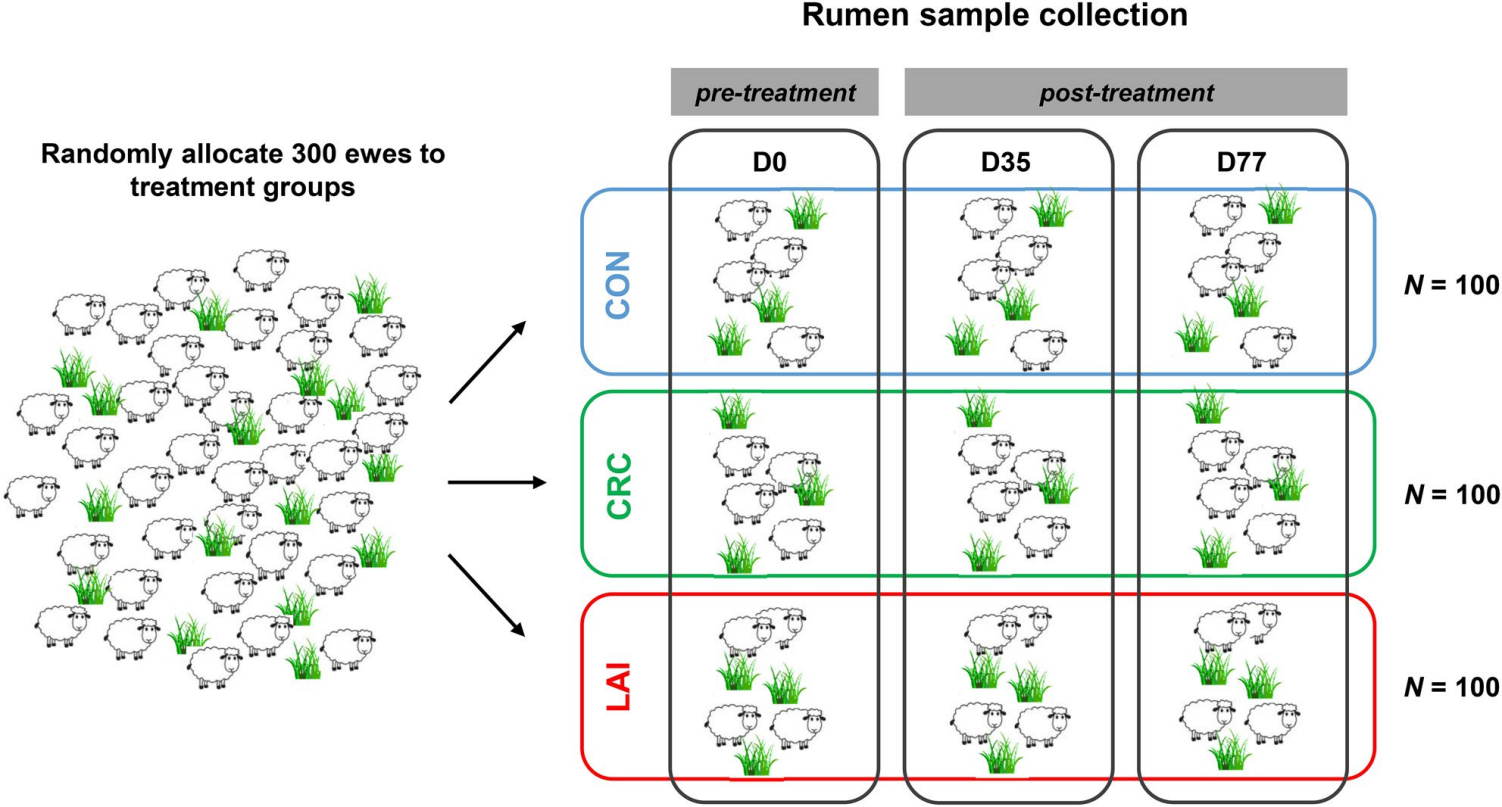
Schematic overview of the study design showing the anthelmintic treatment of sheep and rumen sampling schedule. Three hundred ewes were randomly allocated to the CON, CRC and LAI groups, and rumen content samples were taken just prior to administration of the long-acting anthelmintic treatments on day 0 (D0). The ewes were grazed together in a single mob and rumen contents were sampled again at days 35 (D35) and 77 (D77).

**Figure 2:**
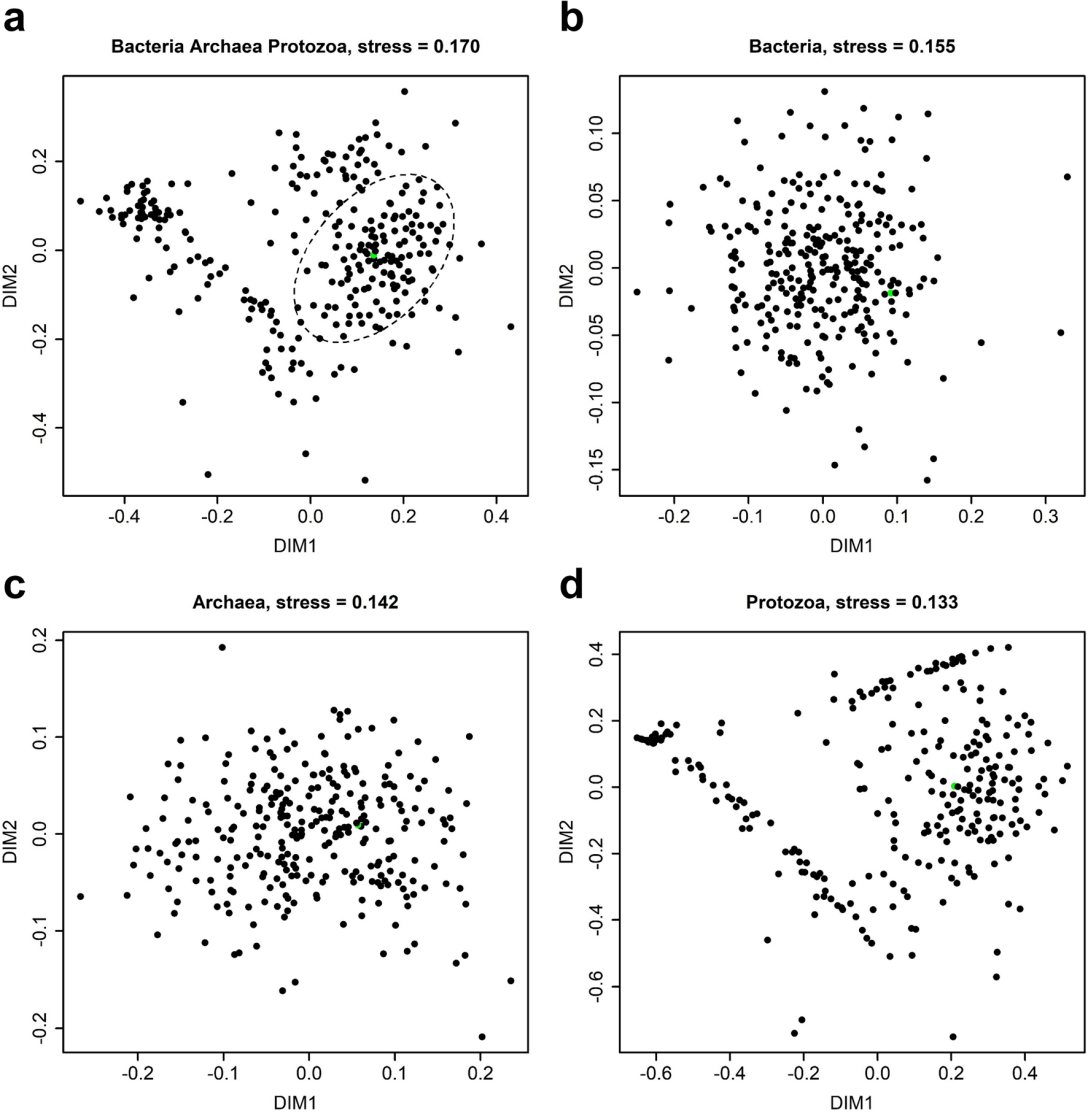
Non-metric dimensional scaling analyses of the rumen bacterial, archaeal and protozoal communities on day 0 prior to administration of anthelmintic treatments. Analyses are based on partial small subunit rRNA gene sequences from (**a**) bacteria (genus level), archaea (genus or lower level) and protozoa (genus level) combined, (**b**) bacteria only, (**c**) archaea only, and (**d**) protozoa only. NMDS dimensions 1 and 2 are denoted DIM1 and DIM2, respectively. NMDS ordination stress values are shown above plots. The data from sheep ID 129 was selected as a reference point within the largest cluster of datapoints (indicated within the ellipse) in panel (**a**), and is shown in green in all panels.

We selected a set of 300 ewes for this study (outlined in Fig. 1) and randomly assigned them to three groups. The ewes in one group were treated with a 100-day rumen in-dwelling controlled release capsule (CRC) containing albendazole and abamectin; those in the second with a long-acting injection (LAI) of moxidectin, given subcutaneously; and the third group was a non-treated control (CON) group. Rumen content samples were taken from all ewes just prior to administration of anthelmintic treatments on day 0, and the bacterial, archaeal and protozoal community structures in these were analysed. An average of 44,214, 11,220 and 11,871 high quality bacterial, archaeal and protozoal partial small subunit rRNA gene sequence reads were generated per sample (Supplementary Table S1). Non-metric multidimensional scaling (NMDS) was used to identify a subset of animals that had similar community structures at the start of the trial, which would allow subsequent divergence of the communities due to the anthelmintic treatments to be more readily detected. The starting rumen microbial communities showed clear segregation, particularly along the first dimension of the NMDS analysis (Fig. 2a), that did not appear to correlate with ewe age, liveweight, body condition or faecal egg count scores (Supplementary Fig. S1). Further analyses of each microbial group separately showed that the bacterial and archaeal communities in these individuals were not clearly differentiated but formed a relatively homogeneous cluster (Fig. 2b,c). In contrast, the protozoal communities (Fig. 2d) could generally be separated into five main clusters (Supplementary Fig. S2) that appeared to drive the clustering pattern observed when the data from all three microbial groups were combined (Fig. 2a). Each protozoal cluster had a distinct community structure that had differing proportions of the dominant protozoal taxa *Entodinium*, *Epidinium* and *Eudiplodinium* (Supplementary Fig. S2).

The largest group of animals with similar microbiota compositions was represented by the cluster on the right of Fig. 2a. Sheep from within this cluster also had good representation of multiple dominant protozoal taxa (*Epidinium*, *Eudiplodinium*, *Entodinium* and *Anoplodinium*/*Diplodinium*; Supplementary Fig. S2) and sheep ID 129 was spatially central to this group (Fig. 2a). The 36 animals in each of the three treatment groups, whose rumen microbiomes had the smallest Bray–Curtis dissimilarities to that of sheep ID 129, and for which a complete set of metadata and rumen samples were available, were selected for further analysis (Supplementary Fig. S3). This gave us a set of 108 ewes with similar starting microbiome compositions in which we could identify changes due to the CRC and LAI treatments. The liveweights and body condition scores of the 108 selected ewes over the 77 day period did not differ among treatments (Supplementary Table S2).

### Effect of anthelmintic treatments on rumen microbial community diversity

An average of 40,735 bacterial, 8057 archaeal and 7660 protozoal high quality sequence reads per sample of partial small subunit rRNA gene sequences were amplified from the 108 selected ewes on days 35 and 77 (Supplementary Table S1). In addition, we analysed fungal communities belonging to the *Neocallimastigomycota* by sequencing internal transcribed spacer region 1 (ITS1) amplicons for all 108 ewes from the samples collected at days 0, 35, and 77, which yielded an average of 59,595 reads per sample (Supplementary Table S1).

There were some differences in the alpha diversities of the microbial communities, both in richness (as Observed species and Chao1 index) and evenness (as Shannon index) (Supplementary Fig. S4). The largest impact was observed in the archaeal community, where the CRC group at day 77 showed significant increases in diversity compared to the CON group by all measures tested (Supplementary Fig. S4b; Welch’s *t* test, *P* = 0.0017 for Chao1, *P* = 0.0030 for Observed species, *P* = 0.00028 for Shannon index). At day 35, the Shannon index for the CRC group was also significantly greater than that of the CON group. The bacterial community exhibited a decrease in diversity at day 77 for the Shannon index only (Welch’s *t* test, *P* = 0.028; Supplementary Fig. S4a). Differences between the treatment and control groups were not observed for the protozoal communities (Supplementary Fig. S4c). Differences in diversity (Shannon index) varied for the fungi, where the increase in Shannon index for the LAI group at day 77 was highly significant (Welch’s *t* test, *P* = 0.0035; Supplementary Fig. S4a), and there was a significant increase in the Observed species index for the CRC group at day 35 (Welch’s *t* test, *P* = 0.036), but not at day 77 (Supplementary Fig. S4d).

### Changes in community composition

**Figure 3:**
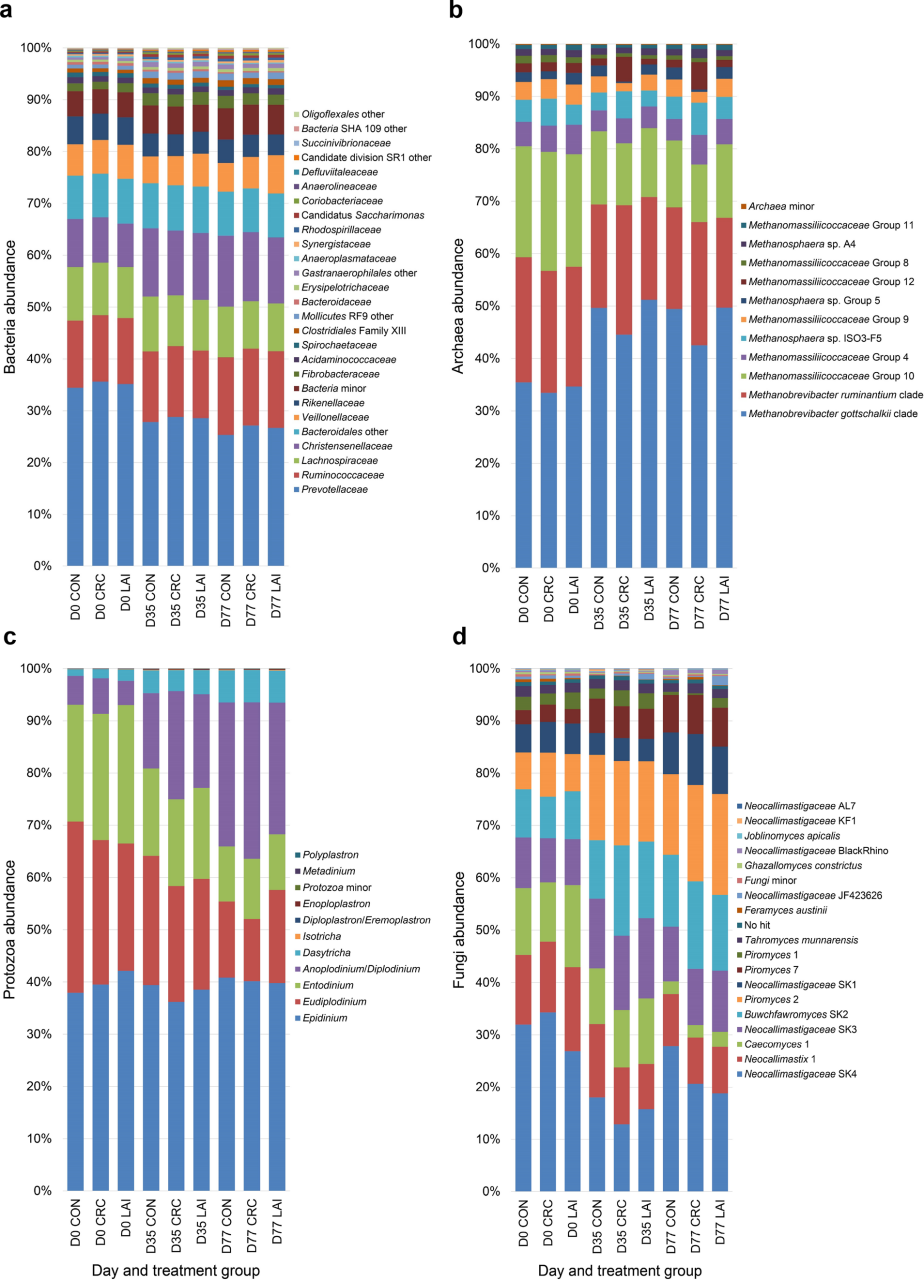
Sheep rumen bacterial, archaeal, protozoal and fungal community compositions between treatment groups at days 0, 35 and 77. Stacked bar charts showing sheep rumen (**a**) bacterial family, (**b**) archaeal genus (or lower level), (**c**) protozoal genus, and (**d**) fungal species level taxa community compositions over time for each treatment group (n = 36). D0, D35, and D77 indicate the sampling days (see Fig. 1).

The communities exhibited only small differences in taxon abundances with respect to treatment at each timepoint, with much larger differences observed between sampling days (Fig. 3). However, the most obvious shifts involving dominant taxa were within the Archaea, where the *Methanobrevibacter gottschalkii* clade decreased in relative abundance in the CRC treatment group compared to the CON group, while the *Methanobrevibacter ruminantium* clade increased. The dominant fungal taxa, *Neocallimastigaceae* SK4 and *Neocallimastix* 1 decreased in relative abundance in the CRC and LAI groups, compared to the CON group, while *Buwchfawromyces* SK2 increased (Fig. 3).

**Figure 4:**
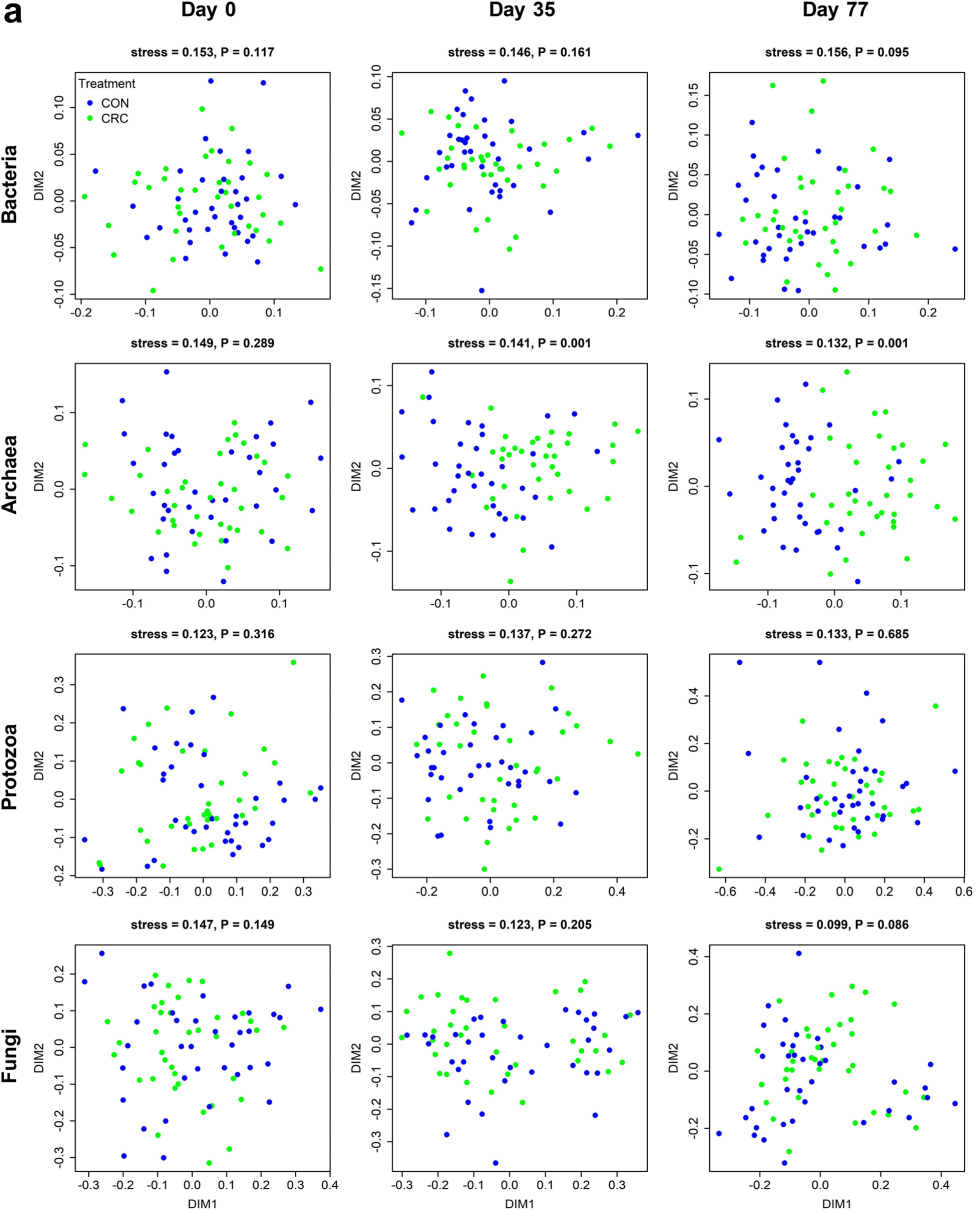

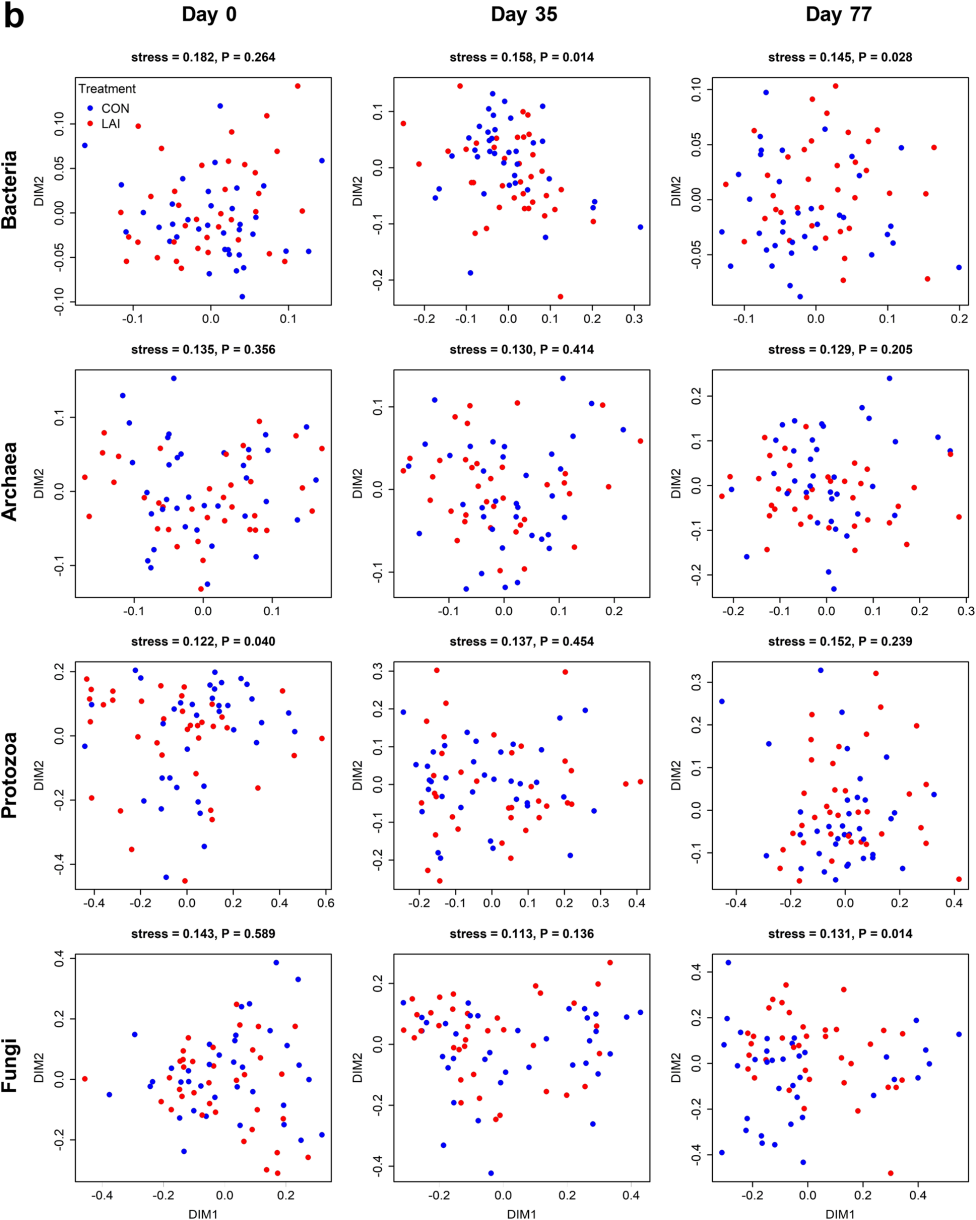
Non-metric dimensional scaling plots showing the impact of the CRC and LAI treatments on sheep rumen bacterial, archaeal, protozoal and fungal communities over time. Plots comparing rumen microbiota from sheep on (**a**) CON and CRC treatments, and (**b**) CON and LAI treatments. NMDS ordination stress values and ANOSIM *P* values comparing microbiota between treatments are shown above plots.

NMDS analysis (Fig. 4) showed that the composition of the bacterial communities of the CRC-treated ewes did not result in significant changes compared to the control group (Fig. 4a). However, the LAI-treated ewes differed from those of the control group at days 35 and 77, and these differences were significant by analysis of similarities (ANOSIM) testing (Fig. 4b; *P* = 0.014 and *P* = 0.028, respectively). The archaeal communities of the CRC treated ewes clearly differed from those of the control group (Fig. 4a; ANOSIM *P* = 0.001 for both days 35 and 77), while no differences were detected due to the LAI treatment (Fig. 4b). The protozoal community structures did not appear to be affected by either of the anthelmintic treatments (Fig. 4). By day 77, the rumen fungal communities in the LAI-treated ewes significantly differed from those of the control group (Fig. 4b; ANOSIM *P* = 0.014).

**Figure 5:**
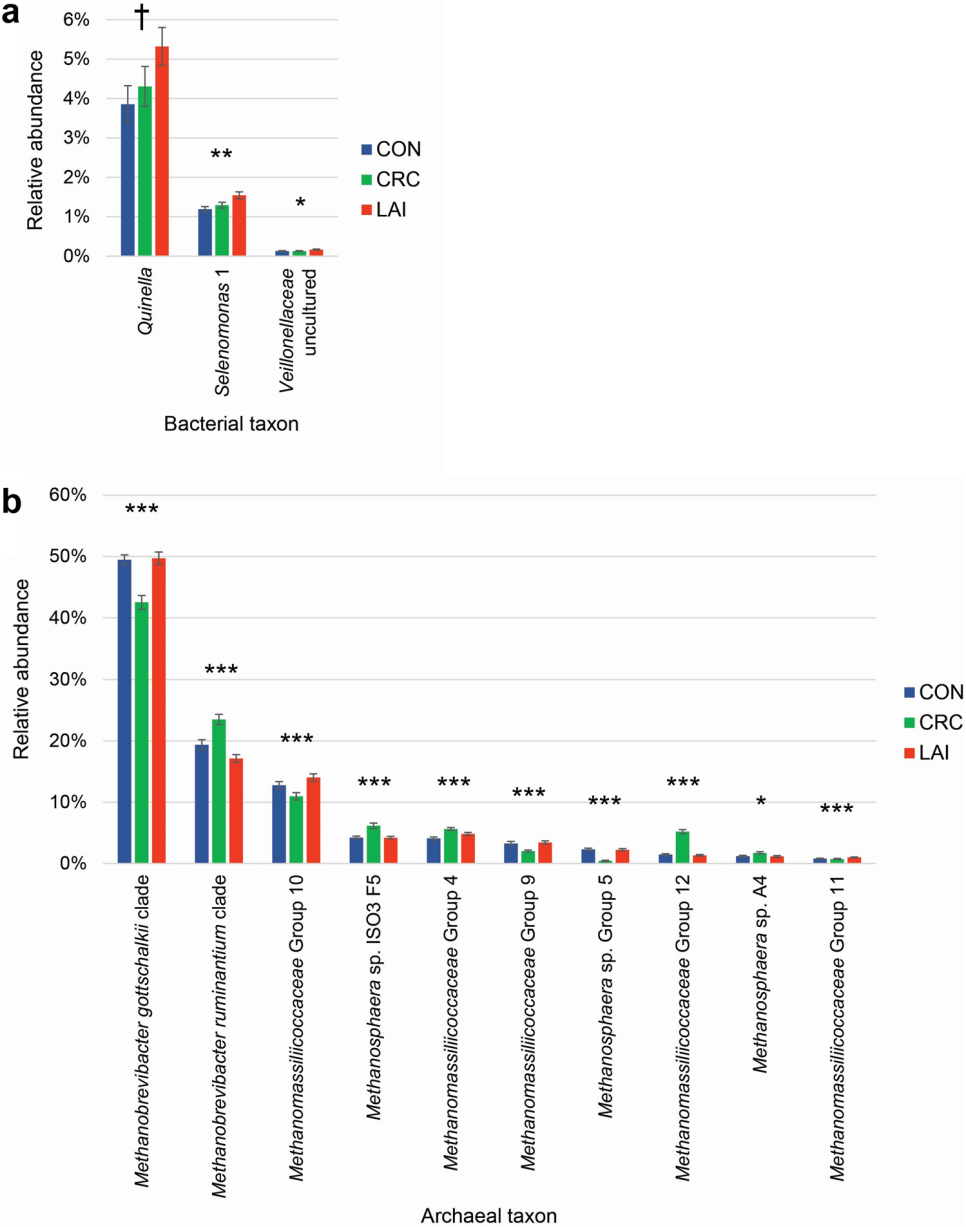
Effects of anthelmintic treatments on members of the family *Veillonellaceae* and methanogenic archaea. Bar charts displaying selected taxa that were differentially abundant (Supplementary Table S4) due to the CRC and LAI treatments at day 77 of the trial. Bars and error bars represent the means and standard error of the mean relative abundances for (**a**) genus-level taxa in the *Veillonellaceae* that were substantially increased by the LAI treatment, and (**b**) archaeal species-level taxa impacted by the CRC treatment. Results of one-way ANOVA testing are shown as ***P < 0.001; **P < 0.01; *P < 0.05; ^†^*P* = 0.096; n = 36 per treatment.

More taxa had significantly different relative abundances, compared to the controls, on day 77 than on day 35, suggesting a slowly building impact of the treatments (one-way analysis of variance to test for treatment effects, Supplementary Table S3 and Supplementary Table S4). The bacterial community response to the LAI treatment was most notably associated with changes in the relative abundances of members of the family *Veillonellaceae*, with 20.6–38.0% increases in mean relative abundance of *Selenomonas* 1, uncultured *Veillonellaceae*, *Quinella* and *Anaerovibrio* compared to the CON group at day 77 (Fig. 5a). Otherwise, the majority of the significant changes for the bacterial taxa tended to be around 10–15% decreases in relative abundances that were associated with lowly-abundant taxa, mainly present at < 0.5% each (Supplementary Table S4). In contrast, the majority of the archaeal taxa displayed significant changes in relative abundances (Supplementary Table S4, Fig. 5b). Notably, *Methanomassiliicoccaceae* Group 12 was, on average, 255% in greater abundance in the CRC group compared to the CON group on day 77 (5.22% ± 0.33% vs 1.47% ± 0.15% (mean ± standard error of the mean), respectively). *Methanosphaera* sp. Group 5 showed an 80.5% reduction in mean relative abundance of the CRC group compared to the CON group (0.45% ± 0.09% vs 2.31% ± 0.20%, respectively). The dominant archaeal taxa also underwent statistically significant changes in relative abundance, with the *Methanobrevibacter gottschalkii* clade decreasing from 49.5% ± 0.08% to 42.5% ± 1.1%, while the *Methanobrevibacter ruminantium* clade increased from 19.4% ± 0.8% to 23.5% ± 0.8% (Fig. 5b). There were few changes in abundance among the protozoal taxa (Supplementary Table S3). Among the fungi, *Neocallimastigaceae* SK4 decreased in abundance in both treatment groups (20.6% ± 2.1% and 18.8% ± 1.4% for CRC and LAI, respectively, compared to 27.8% ± 2.8% in CON). In contrast, among the most abundant fungal taxa, abundances of *Piromyces* 2 and *Neocallimastigaceae* SK1 both tended to increase relative to CON (*P-*values of 0.07 and 0.09, respectively) (Fig. 5).

### Co-abundance relationships between rumen microbial groups

Rumen microbes perform a range of functional roles within the rumen ecosystem, such as fibre degradation, protein degradation, fermentation, hydrogen utilisation and methane formation, that together facilitate the conversion of feed to energy substrates for the host, as well as other rumen microbes. A key feature of community function is the strong metabolic interaction between different microbial groups, often in the form of metabolite cross-feeding, which may be undertaken via intimate microbe-microbe interactions, such as methanogen endosymbionts of protozoal hosts^14^. We examined co-abundance relationships between microbial taxa within the treatment groups to gain insights into possible interactions between community members and whether the anthelmintic treatments had impacted these relationships. We generally observed similar relationships between taxa in the CON, CRC and LAI treatments (Supplementary Fig. S5), with the largest Spearman correlations between taxa observed within the CON group (Supplementary Fig. S5a), and the intensity of correlations lessened for the groups that received anthelmintic treatments (Supplementary Fig. S5b and S5c). The two largest network clusters in the CON group contained members of the *Lachnospiraceae*; and had members of the *Firmicutes* (including *Christensenellaceae* R-7) that were negatively correlated with *Prevotella* 1. Within the CRC treatment, these two networks converged to form a single larger network, where the abundance of *Prevotella* 1 negatively correlated with *Blautia* of the *Lachnospiraceae* (as compared to with *Clostridiales* Family XIII members as seen in the CON samples). In addition, co-occurrence of the *Methanosphaera* taxa Group 5 and A4 were apparent among the CRC group, which was the only treatment to feature co-occurrence of archaeal taxa. The LAI samples revealed relationships between various members of the *Ruminococcaceae* and fungal taxa, where *Ruminococcaceae* UCG-002 was negatively correlated with *Caecomyces* 1, but positively correlated with *Neocallimastigaceae* SK1. Across all treatments, *Buwchfawromyces* was negatively correlated with *Neocallimastix* 1 and *Joblinomyces apicalis*.

## Discussion

The aim of this study was to determine the impacts of broad spectrum and long-acting anthelmintic products on the composition of the rumen microbiota of grazing sheep. We found that there were clear differences in rumen microbial community structures over the course of the 77 days of the trial. These differences were largely attributed to the changes expected in pasture composition over the same time period. At the two timepoints after treatment began, the two anthelmintic products resulted in relatively modest differences in the compositions of the communities compared to the controls at those same times. This result was somewhat unexpected, given the broad spectra of activities that these active compounds are known to exhibit, though we are unaware of any previous studies that have looked at the effect of these compounds on the rumen microbiota. A few studies have examined the activities of albendazole and moxidectin on lower gut microbiota in situ, and only small changes in microbial diversity and composition have been reported^11,15,16^, although these studies have focused analysis on the bacterial community only.

The CRC treatment, which slowly releases albendazole and abamectin (an avermectin) directly into the rumen, resulted in the largest impact on the rumen microbiota in the present study, and this affected both the diversity and composition of the archaeal community. Significant increases in both archaeal taxon richness and evenness were observed after 77 days. Moreover, differences in the relative abundances of nine archaeal genera (members of the main rumen archaeal classes *Methanobacteria* and *Thermoplasmata*)*,* were significant compared to the control. However, the differences were opposing relative changes in the abundance of *Methanomassiliicoccaceae* Group 12 and *Methanosphaera* spp., both of which grow using hydrogen plus methyl groups to form methane. Similarly opposing changes in the relative abundance of two different clades of the genus *Methanobrevibacter*, both of which grow using hydrogen plus carbon dioxide to form methane, were observed. It is unlikely that these changes would materially alter the amount of methane formed from these treated sheep. The CRC treatment did not alter the alpha diversity of the bacterial, protozoal or fungal communities, and only few changes to the relative abundances of minor taxa were observed, where the majority of these were members of the *Clostridiales*. As the CRC treatment produces a direct and sustained release of albendazole and abamectin into the rumen via a rumen in-dwelling capsule, the microbiota were directly exposed to the anthelmintic compounds for an extended period. Thus, it was surprising that we did not detect larger impacts on the more abundant members of the microbial community. Changes to the fungal community might have been expected, given that albendazole has antifungal activities, and avermectins have been shown to inhibit chitin synthesis^7^. However, anaerobic fungi occupy a specialised niche in the gut, and their physiology differs considerably to that of aerobic fungal pathogens. For example, the anaerobic fungi lack mitochondria, but possess hydrogenosomes^17^, and it is conceivable that the mode of action of albendazole, and potentially of abamectin, is not effective against the rumen anaerobic fungi. Alternatively, the dosage released into the rumen may have been too low to cause substantial changes to the fungal communities. To our knowledge, the effects of abamectin on rumen or lower gut microbiota have not previously been examined. Albendazole has been used to treat helminth infections in the hindgut. In one study, this was associated with decreases in the abundance of *Clostridiales* in the human gut^18^, while in another study increases in *Clostridiales* were observed^16^. The hindgut, in contrast to the rumen, has complex interactions between the immune system, gut microbiota, anthelmintic treatments, and activities and clearance of parasites, which are thought to contribute the observations seen in these studies^16,18^. We are therefore cautious in comparing our observations of anthelmintic compounds on the rumen microbiota to studies on the hindgut microbiota, because parasites do not colonise the rumen and their impact in the rumen is probably minimal as they transit through. Similarly, the impact of the immune system on the rumen microbiota is also minimal, compared to in the hindgut. Moreover, differences in the pharmacokinetics of the compounds, and differences in the methods of their administration (e.g., oral, topical, subcutaneous, intraperitoneal) may alter their effects on the microbiota in the rumen and the hindgut. In this study, it is possible that the two methods of anthelmintic administration (controlled release capsule and subcutaneous injection) contributed to observed treatment differences on the rumen microbiota. Further studies that control for administration would need to be undertaken to directly compare the effects of the anthelmintic compounds.

The LAI treatment, in contrast to the CRC treatment, affected the bacterial and fungal communities, resulting in reductions in bacterial diversity and increases in fungal diversity. Detectable changes in community structure were also apparent, with members of the family *Veillonellaceae* in particular being substantially enriched for. The cause for the enrichment of *Veillonellaceae* by the moxidectin treatment is not known, but these are likely to be propionate-forming bacteria^19^ that do not degrade complex plant polymers. Whether the increases in their abundance are associated with more energetically-favourable fermentation towards more propionate is not known^20^. However, the LAI treatment did not result in significant changes to liveweight gain and body condition score compared to the CRC treatment groups, or the control for the animals examined. There was a significant decrease in the relative abundance of *Neocallimastigaceae* SK4 by both treatments. SK4 was the most abundant rumen fungal genus detected, but these organisms are as-yet poorly understood. There were also increases in the relative abundances of *Piromyces* 1 and *Piromyces* 2 in the LAI treatment group. Knowledge of the diversity and structure of rumen fungal communities is more limited than for the bacteria, archaea and protozoa. Efforts to improve the taxonomy of the *Neocallimastigomycota* have increased in recent years, with seven new genera being described recently^21^. The application of modified primers targeting the ITS1 region to capture these new taxa, together with the most recent update of the anaerobic fungi database, have enabled the detection of genera such as *Buwchfawromyces*^22^ (formerly SK2), which was first described from buffalo faeces, and was revealed among the most abundant rumen fungal taxa detected in this study. The impacts on rumen fermentation and function, if any, of the changes in the bacterial and fungal communities due to the LAI treatment are not known. However, positive and negative correlations between the abundances of fungal taxa and members of the family *Ruminococcaceae* were apparent, and suggestive of potential synergies and competition between these different taxa for the degradation of fibre. Differences in the archaeal community due to the LAI treatment were not observed, so it is presumed that any impacts to fermentation are minor and did not significantly impact the supply of energetic substrates to the archaea.

Neither anthelmintic treatment tested in this study appeared to impact the rumen protozoal communities. Rumen ciliate protozoa are generally more variable under similar host and diet conditions than the bacteria and archaea^23^, and community types have been defined that are generally dominated by very few taxa, underpinned by complex antagonistic interactions between these^24–26^. In the present study the diversity of the protozoa was largely responsible for differentiating the rumen microbial communities of the 300 ewes at the start of the trial. The protozoal communities appeared consistent with Type B (*Epidinium* and/or *Eudiplodinium*-dominant), and Type O (*Entodinium*-dominant) communities^27^. We selected the ewes from the largest cluster identified by NMDS for subsequent analyses, so that the greatest numbers of ewes with similar starting community compositions would be used to test for potential community differences as a result of anthelmintic treatment. The selected animals initially had varying proportions of *Epidinium*, *Eudiplodinium* and *Entodinium, Anoplodinium/Diplodinium*, but the anthelmintic treatments did not significantly impact the protozoal communities compared to the control group. It is possible that the treatments may have influenced the other protozoal community types that were initially observed (e.g. Supplementary Fig. S2a, clusters 2–5), but were not further examined in this study.

Few strong positive and negative associations were observed in microbial co-abundance networks within the untreated sheep. The general lack of strong association patterns between rumen microbial taxa has been previously observed, and it was suggested that interactions between different functional groups of taxa are flexible and may occur via common pools of metabolites that cross feed from producers to utilisers^23^. The anthelmintic treatments appeared to dampen the strongest correlations, but generally resulted in minor impacts to the networks overall which suggests that functional relationships within the communities largely remained intact.

Recent studies in lambs have reported that infection by gastrointestinal nematodes can affect rumen microbial composition^28,29^, and thus it cannot be ruled out that changes in rumen microbial community structure due to anthelmintic treatment may actually be due to the presence of parasites in the CON group^30^ rather than the anthelmintic treatment itself. In our trial, the untreated ewes were all healthy (e.g., gaining weight and conceiving lambs), and were all presumed to have a low level of parasite infection, where the average faecal egg count observed was less than 140 eggs/gram of faeces^30^. In contrast, much younger animals were used in the studies where changes in the rumen microbiota were observed, and those trials resulted in quite severe infections^28,29^. Correa et al.^28^ infected 10 month old lambs, resulting in severe infections with more than 700 eggs/gram of faeces (epg) in infected groups, and El-Ashram et al.^29^ dosed 3 month old parasite-naïve lambs with 5000 *Haemonchus contortus* L_3_ larvae. These levels of infection are likely to impact the overall physiology of the animal (e.g., immune response, rumen pH and other parameters) that may in turn affect the rumen microbiome. Given that we observed very few differences in microbial taxon relative abundances between infected (CON) and parasite-free animals (LAI and CRC) (Supplementary Tables S3 and S4), this suggests that parasite presence in the CON group had minimal, or no, impact on the rumen microbiome in our study. It is possible that anthelmintic treatment effects will be large if there are resultant changes in parasite load with flow-on effects on the microbiota, rather than having large direct impacts on the microbes. In addition, given the difficulties in maintaining parasite-free and parasite-infected animals in a field environment, there was no practical alternative to using the approach we had undertaken with the number of animals used. To further investigate the role of the anthelmintic treatments on the rumen microbiome in the absence of parasites, controlled animal studies or in vitro investigations would need to be undertaken.

Few changes in the microbial community structures were apparently due to the anthelmintic treatments, and it is not known whether these impacted the metabolism of the rumen. Given that ewe production benefits were largely temporary^30^, the previously observed changes in liveweight associated with the anthelmintic treatments were most likely a result of a temporary clearance of parasites, rather than changes to the microbiome and rumen microbial metabolism. The observation that differences in microbiota were attributable to the anthelmintic treatments does emphasise the multitude of impacts that administration of these products can have, similar to the trace element effects reported by Miller et al.^4^, over and above their expected effects of killing parasitic worms. Deeper analysis of community function (e.g., at the transcriptional, translational or metabolite level), and interactions with host phenotype and genotype, may provide a more comprehensive view of any impacts of the treatments on rumen microbial community function and contribution to host physiology.

## Conclusions

To our knowledge, this study represents the first investigation into the impact of long-acting anthelmintic treatments on the rumen microbiota of sheep with a very low parasite burden. There were few changes in the relative abundances of bacterial and fungal taxa, and these varied depending on the anthelmintic treatment used. The CRC treatment affected most of the archaeal taxa, though the absolute differences in their relative abundances were low. The relationship between these changes, rumen fermentation, and ewe performance, including methane production, are not known and would require further investigation. However, our results further illustrate the complexity of effects that administration of anthelmintic compounds with broad spectra of activity can have on ruminant animals.

## Methods

### Trial design and sample collection

The trial (Fig. 1) was conducted on a sheep and beef station near Rotorua in the North Island of New Zealand and is described in detail by Leathwick et al.^30^. In brief, a mob of approximately 800 mixed aged, Poll Dorset Texel cross ewes, grazing a ryegrass-based pasture, were visually assessed for body condition (‘light’, ‘medium’ and ‘heavy’) and, within each body condition group, were randomly allocated to anthelmintic treatment groups to ensure a range of body conditions were represented within each treatment group. The three treatments examined in this study were a 100-day CRC that contained 4.62 g albendazole, 160 mg abamectin, 24 mg selenium, and 120 mg cobalt (Bionic, Merial New Zealand Limited, Auckland, New Zealand) inserted directly into the rumen using an applicator gun; a LAI containing 20 g/L moxidectin, injected subcutaneously at the manufacturer’s recommended rate of 1 mg/kg bodyweight (Cydectin Long Acting, Zoetis New Zealand Limited, Auckland, New Zealand); and an untreated CON group. The release kinetics of moxidectin into the rumen via the LAI are not known. However, the manufacturer reports that this product provides protection against internal parasites for up to 112 days, and has a milk withhold period of 185 days. Therefore, the active is likely still being released from the deposition site for the 77 days of sampling in this experiment. Through subcutaneous injection, moxidectin concentrations were much greater in the abomasal tissue and mucosa than in abomasal content^31^, and it is reasonable to expect a similar situation in the rumen. Moreover, moxidectin is highly stable in sheep rumen contents, where it extensively associates with the solid phase of the digestive contents^32^.

Each animal was then weighed and manually assessed for body condition score. By approval of the AgResearch Grasslands Animal Ethics Committee (Application number 14338), faecal samples for faecal nematode egg count, and rumen content samples of approximately 30 mL, obtained by stomach intubation, were taken. If rumen samples could not be obtained after two attempts at intubation, the sample was noted as missing. Samples were immediately frozen at −20°C and stored until required.

Treatments were then administered to animals according to tag numbers. Animals with tags 1–100 received the CRC treatment, tags 101–200 received the LAI treatment, and tags 201–300 were the CON group. The sheep were managed as a single flock on ryegrass-based pasture and were reassessed for liveweight and body condition score on days 35 and 77^30^, when rumen content samples were also obtained.

### Parasitology

Faecal egg count data was determined using the McMaster method^33^, with modifications as previously described^30^.

### Rumen microbial community analysis

The frozen rumen content samples were freeze-dried, ground, homogenized and total DNA was extracted using a bead-beating based method according to established protocols^23,34^. The bacterial and archaeal 16S rRNA gene regions and ciliate protozoal 18S rRNA gene regions were PCR-amplified^35^, and pooled together for sequencing as described by Kittelmann et al.^36^. Amplification and sequencing of the anaerobic fungal internal transcribed spacer 1 (ITS1) regions was undertaken separately to that for the bacterial, archaeal and protozoal markers. Primers MN100F and MNGM2R^37^ were modified to take into account all 18 anaerobic fungal genera described to date, including newly defined anaerobic fungal taxa^21^. The fungal-specific regions of modified primers, MNGMR2: 5′-CTGCGTTCTTCATCGTTGCG-3′ and MN100F2: 5′-TCCTACCCTTTGTGAATT-3′, are predicted to amplify regions approximately 130–304 bp in length^38^. All primer sequences are shown in Supplementary Table S5.

Pools of bacterial, archaeal and protozoal amplicons, or fungal ITS1 amplicons, were sequenced using Illumina MiSeq 2 × 250 base PE V2 runs at the Massey Genome Service (Palmerston North, New Zealand). Quality control of the raw sequence data used BWA alignment at a 0.01 cutoff with the BWA trimming option in SolexaQA++^39^. Read 1 data were processed and analysed using QIIME software V1.9.1^40^ with operational taxonomic unit (OTU) picking performed at 99% sequence identity prior to aggregation at the desired taxonomic level for each microbial group after OTU taxonomic assignment. Representative sequences for each OTU were assigned taxonomic strings by BLAST^41^ using the blastn algorithm, with megablast option, and an e-value cutoff of 0.001, against an in-house database comprising the SILVA V123 database^42,43^, which contains updated taxonomy for the rumen bacteria; and archaeal 16S rRNA gene and protozoa 18S rRNA gene databases as previously described^44,45^. The fungal ITS1 sequences were assigned based on V3.5 (release February 2020; https://anaerobicfungi.org/tools/) of the fungal database by Koetschan et al.^46^. Relative abundances of microbial groups are shown in Supplementary Table S1.

### Statistical analyses

The microbial community composition data were expressed as relative proportions of taxa within each microbial group (bacteria, archaea, protozoa, fungi), and analysed using R V3.6.1^47^ implemented in RStudio V1.2.1335 (http://www.rstudio.com/). Microbial taxa were filtered to include only those with mean relative abundances of >0.1% for bacteria at the genus level and archaea at the genus (or lower) level, and > 0.01% for protozoa genus level. All minor taxa within the bacteria, archaea and protozoa were grouped together as Bacteria minor, Archaea minor and Protozoa minor. Filtering was not required for the rumen fungi at the species level as the mean relative abundances of all taxa were > 0.1%. Statistical analyses were performed on the relative abundance data at these taxonomic levels.

Welch two sample *t* tests and one-way ANOVA testing was performed using base functions in R. Least significant difference post hoc analysis of ANOVA results was performed using the R package, agricolae^48^. NMDS, performed on Bray–Curtis dissimilarity matrices^49^, and stress values, were calculated using the VEGAN R package^50^, and the main clusters were identified by visual inspection. ANOSIM^51^ performed using the VEGAN R package with the default settings (including Bray–Curtis distance calculations), was used to test the null hypothesis that the similarity between the different subsets of the animals tested is greater than or equal to the similarity within the subsets. Positive ANOSIM R values suggest greater similarity within subsets than between, values close to zero suggest no difference between within subset and between subset similarities, and negative values suggest greater similarity between subsets than within.

Alpha diversity was assessed using Chao1, Shannon index and Observed species implemented in QIIME V1.9.1 using read count data with a minimum read counts per sample of 9000 for bacteria, 2200 for archaea, 1900 for protozoa, and 3000 for fungi.

Spearman correlations were generated using the rcorr function from the Hmisc package for R^52^. Networks were generated from the correlation matrix using the igraph package for R^53^, with correlation (r) cut offs of > 0.6 and *P* < 0.05. Networks were visualised in Cytoscape 3.8 using a prefuse force directed layout^54^.

Ewe liveweight and body condition score changes over time were analysed using a linear mixed model (REML) which included anthelmintic treatment and time as factors, ewe age, initial (D0) liveweight, and body condition score as covariates, along with the interaction terms for treatment × time, treatment × body condition score and time × body condition score^27^. The ewe liveweight, body condition score and age covariates were centred by subtracting every value by their respective means.

### Ethics declarations

This study involves the use of animals and was approved by the AgResearch Grasslands Animal Ethics Committee, application number 14338. All experiments were performed in accordance with the approval guidelines and regulations, and this study complies with the ARRIVE guidelines (http://www.nc3rs.org.uk/page.asp?id=1357).

## Supplementary Information

Supplementary Information

## Data Availability

The datasets supporting the conclusions of this article are available in the NCBI sequence read archive, BioProject PRJNA683928 in https://www.ncbi.nlm.nih.gov/sra.
