# Supplementary material for: Effects of long-acting, broad spectra anthelmintic treatments on the rumen microbial community compositions of grazing sheep
Source: Sci Rep. 2021 Feb 15;11:3836. doi: 10.1038/s41598-021-82815-y (PMC7884727; doi:10.1038/s41598-021-82815-y)
Supplement: Supplementary file 1 — Supplementary Information [file 41598_2021_82815_MOESM1_ESM.pdf]

Supplementary Information

Effects of long-acting, broad spectra anthelmintic treatments on the rumen microbial community compositions of grazing sheep

Christina D. Moon[1*], Luis Carvalho[1], Michelle R. Kirk[1], Alan F. McCulloch[2], Sandra Kittelmann[3], Wayne Young[1], Peter H. Janssen[1], Dave M. Leathwick[1]

[1]AgResearch Limited, Grasslands Research Centre, Palmerston North, New Zealand

[2]AgResearch Limited, Invermay Research Centre, Mosgiel, New Zealand

[3]Wilmar International Limited, WIL@NUS Corporate Laboratory, Centre for Translational Medicine, National University of Singapore, Singapore, Singapore

*Corresponding author

E-mail: christina.moon@agresearch.co.nz

This file includes:

Supplementary Figure S1. Relationship between ewe age, liveweight, body condition and FEC with the microbiome.

Supplementary Figure S2. Diversity of protozoal community structures.

Supplementary Figure S3. Animal selection for microbiome analysis.

Supplementary Figure S4. Alpha diversity of rumen microbiota by microbial group in response to anthelmintic treatments.

Supplementary Figure S5. Correlations between microbial taxa within treatment groups using combined data from days 35 and 77.

Supplementary Table S1. Sequence read abundances for ewe rumen microbiota.

Supplementary Table S2. Treatment groups means and REML analyses for ewes that were selected for microbial community analysis at D0, D35 and D77.

Supplementary Table S3. ANOVA and least significant difference analysis of relative microbial taxon abundance (as percentage) at day 35.

Supplementary Table S4. ANOVA and least significant difference analysis of relative microbial taxon abundance (as percentage) at day 77.

Supplementary Table S5. Primers used in this study.

Supplementary Figure S1. Relationship between ewe age, liveweight, body condition and FEC with the microbiome.

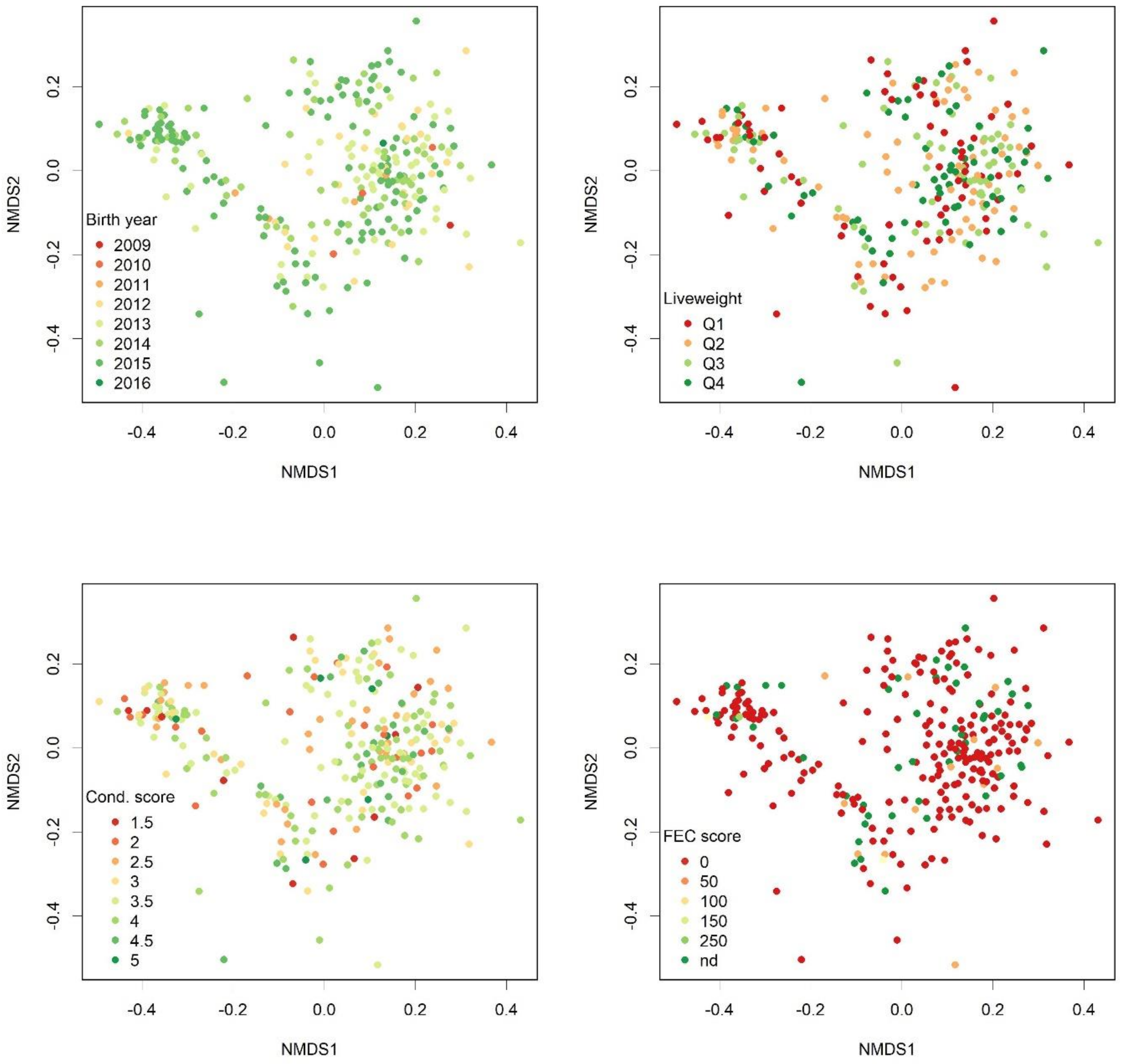

Non-metric dimensional scaling of Bray-Curtis dissimilarities calculated from the bacteria, archaea and protozoa relative abundance data of ewe rumen samples collected on day 0 of the trial. The top-left panel shows data by ewe birth year; top-right shows data by ewe liveweights, classified by quartiles; lower left shows the distribution of ewe body condition scores, and lower right shows the faecal egg count (FEC) scores from samples taken the day of, and prior to, treatment administration.

There were no apparent relationships between these parameters and rumen microbial community structures at the start of the trial.

Supplementary Figure S2. Diversity of protozoal community structures.

a

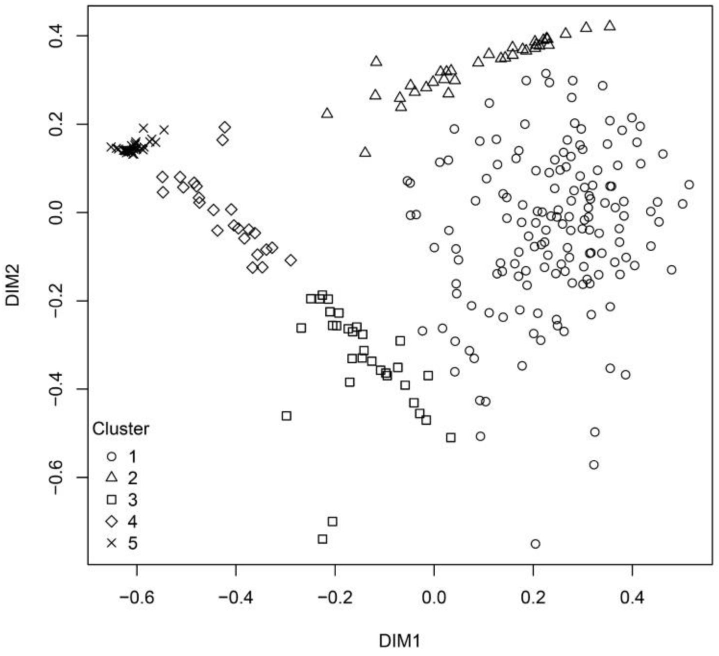

b

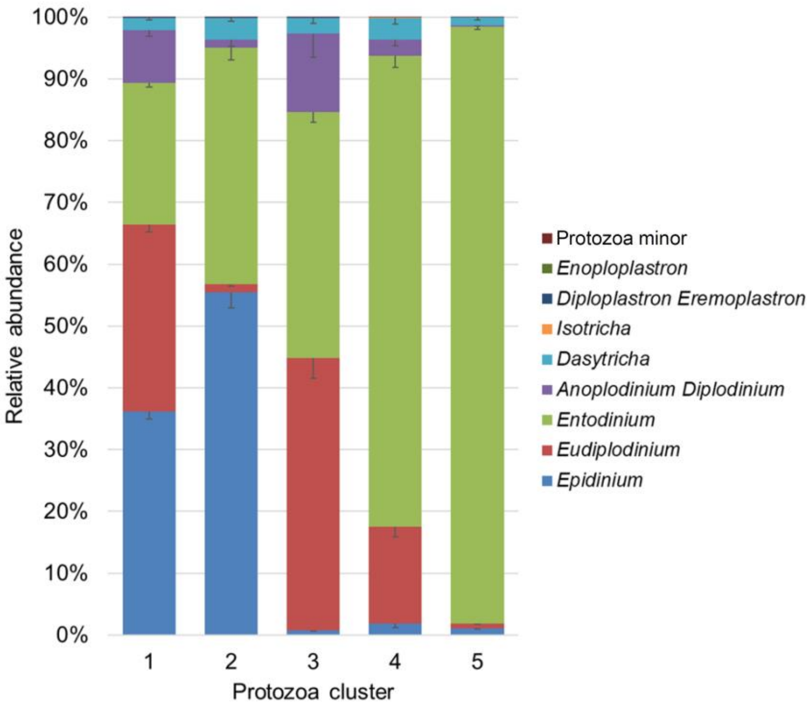

The protozoal microbial communities on day 0 showing (**a**) five main clusters by NMDS analyses with NMDS dimensions 1 and 2 denoted DIM1 and DIM2, respectively, and (**b**) average protozoal community compositions of the clusters, where all clusters appear to be Type B protozoal communities[1].

Supplementary Figure S3. Animal selection for microbiome analysis.

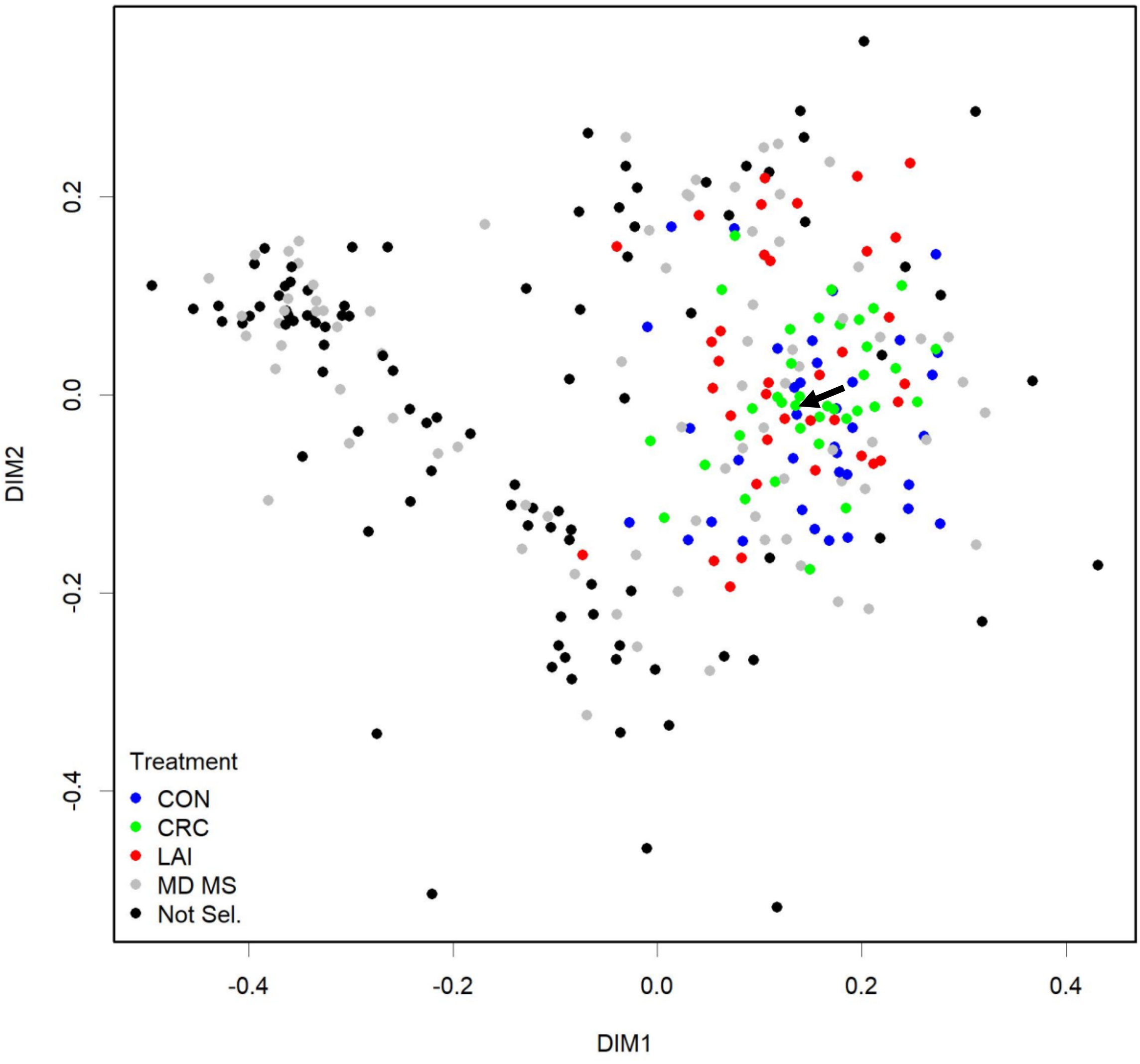

Non-metric dimensional scaling of rumen microbiota data from day 0, based on combined bacterial, archaeal, and protozoal data. Thirty-six ewes per treatment group were selected for further analysis from each treatment group (shown as blue, green and red points), based on their Bray-Curtis dissimilarity to animal ID 129 (arrow). Rankings did not include animals that were missing measurement data or rumen samples (MD MS; grey). All other animals not selected for further analysis (Not Sel.) are shown in black. NMDS dimensions 1 and 2 denoted DIM1 and DIM2, respectively.

Supplementary Figure S4. Alpha diversity of rumen microbiota by microbial group in response to anthelmintic treatments.

a

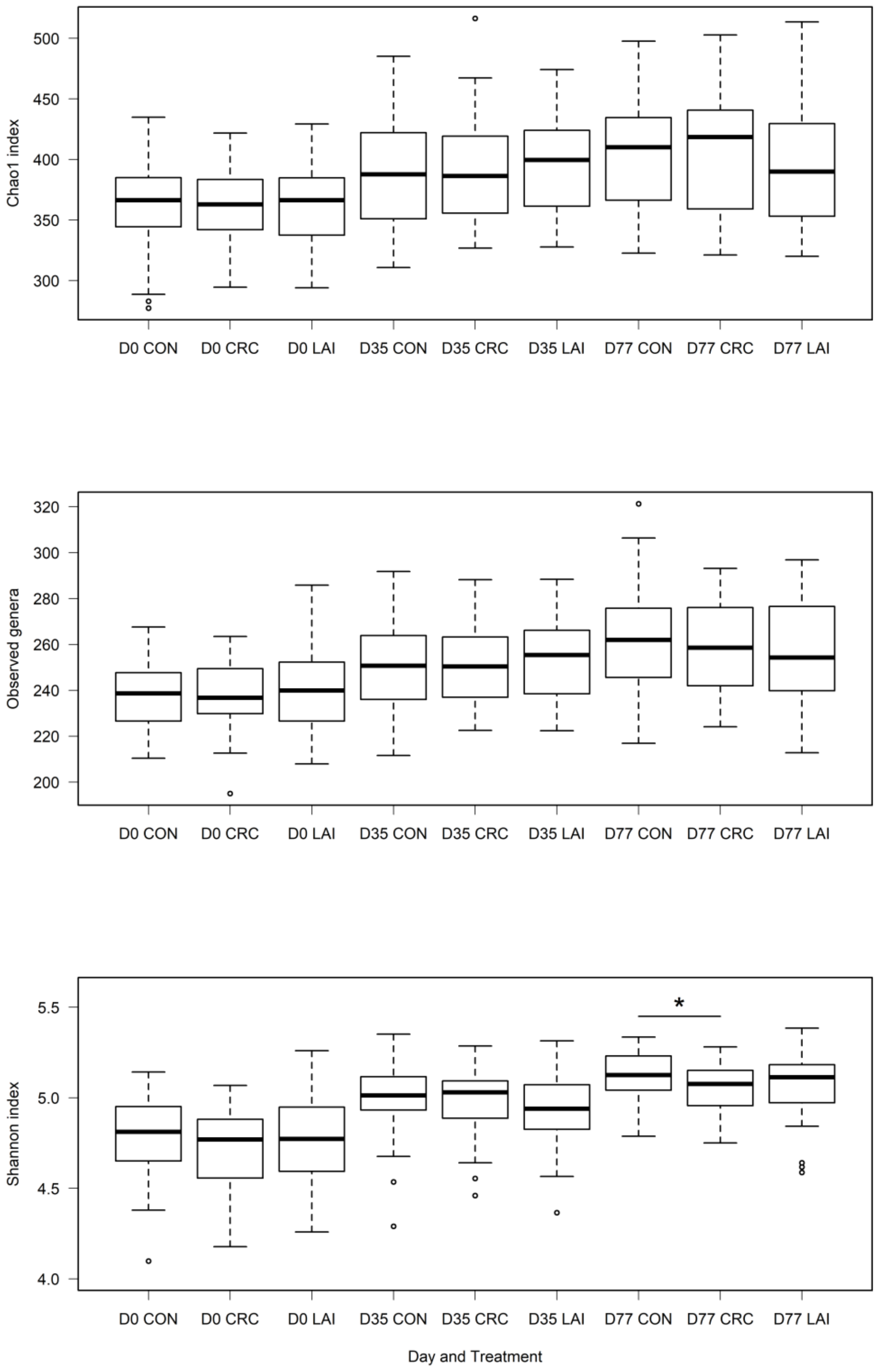

b

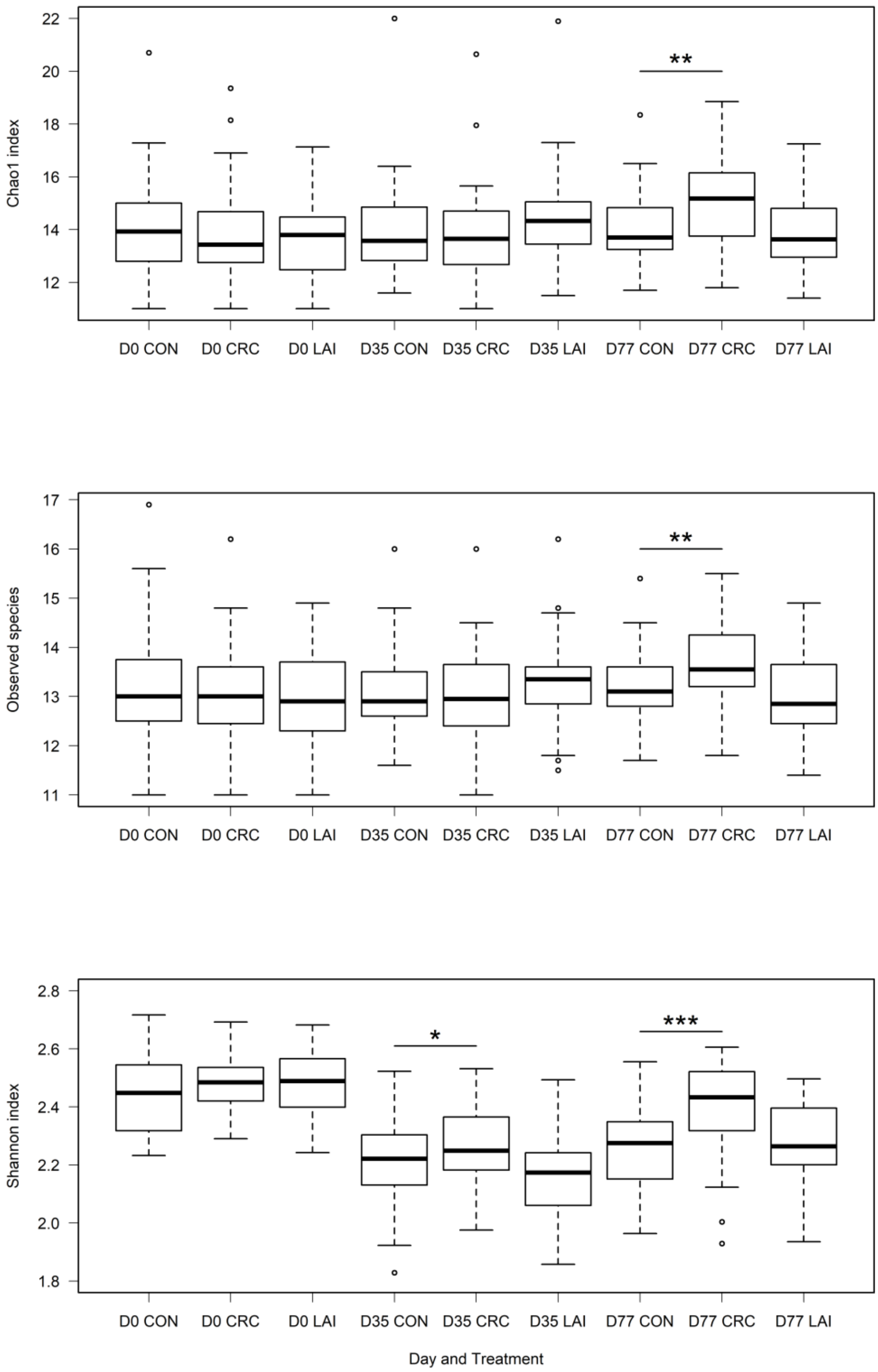
Chao1 index
22
20
18
16
14
12
**
D0 CON
D0 CRC
D0 LAI
D35 CON
D35 CRC
D35 LAI
D77 CON
D77 CRC
D77 LAI
Observed species
17
16
15
14
13
12
11
**
D0 CON
D0 CRC
D0 LAI
D35 CON
D35 CRC
D35 LAI
D77 CON
D77 CRC
D77 LAI
Shannon index
2.8
2.6
2.4
2.2
2.0
1.8
*
***
D0 CON
D0 CRC
D0 LAI
D35 CON
D35 CRC
D35 LAI
D77 CON
D77 CRC
D77 LAI
Day and Treatment

c

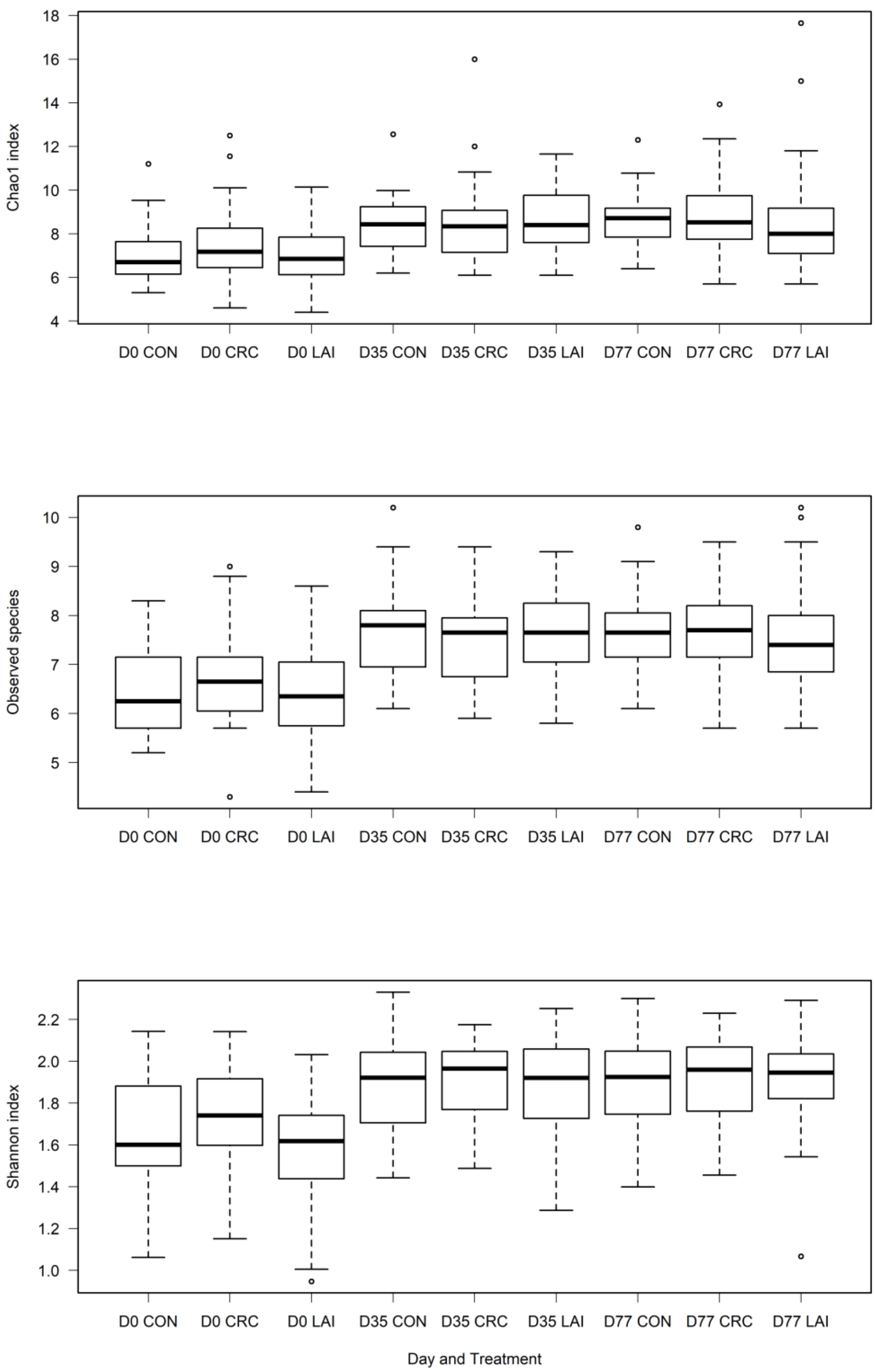
Chao1 index
18
16
14
12
10
8
6
4
D0 CON
D0 CRC
D0 LAI
D35 CON
D35 CRC
D35 LAI
D77 CON
D77 CRC
D77 LAI
Observed species
10
9
8
7
6
5
D0 CON
D0 CRC
D0 LAI
D35 CON
D35 CRC
D35 LAI
D77 CON
D77 CRC
D77 LAI
Shannon index
2.2
2.0
1.8
1.6
1.4
1.2
1.0
D0 CON
D0 CRC
D0 LAI
D35 CON
D35 CRC
D35 LAI
D77 CON
D77 CRC
D77 LAI
Day and Treatment

d

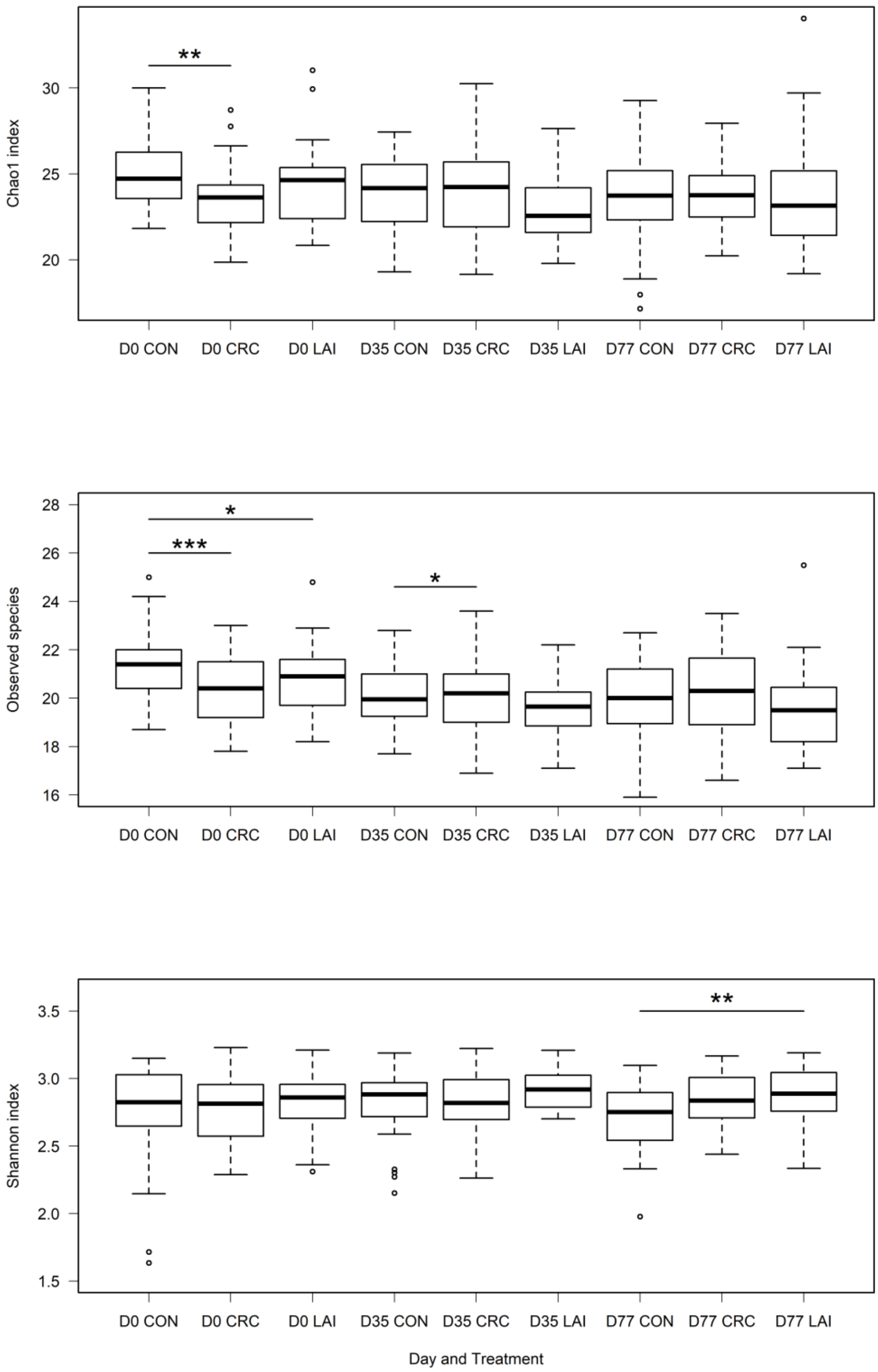

Boxplots showing alpha diversity indices of sheep rumen (**a**) bacterial, (**b**) archaeal, (**c**) protozoal, and (**d**) fungal communities. Each shows the Chao1 index (top), Observed taxa (middle), Shannon Index

(bottom) values for each treatment group and sampling time. Rarefaction was performed at a sampling depth of 9,000, 2,200, 1,900, and 3,000 for the bacteria, archaea, protozoa and fungal datasets, respectively. Welch two sample t-tests were conducted to compare each treatment to the relevant time point control indicated by the extremities of the bar marked with the indicators of significance (*P*-values < 0.05 are denoted by *, *P*-values < 0.01 are denoted by **, *P*-values < 0.001 are denoted by ***).

Supplementary Figure S5. Correlations between microbial taxa within treatment groups using combined data from days 35 and 77.

a

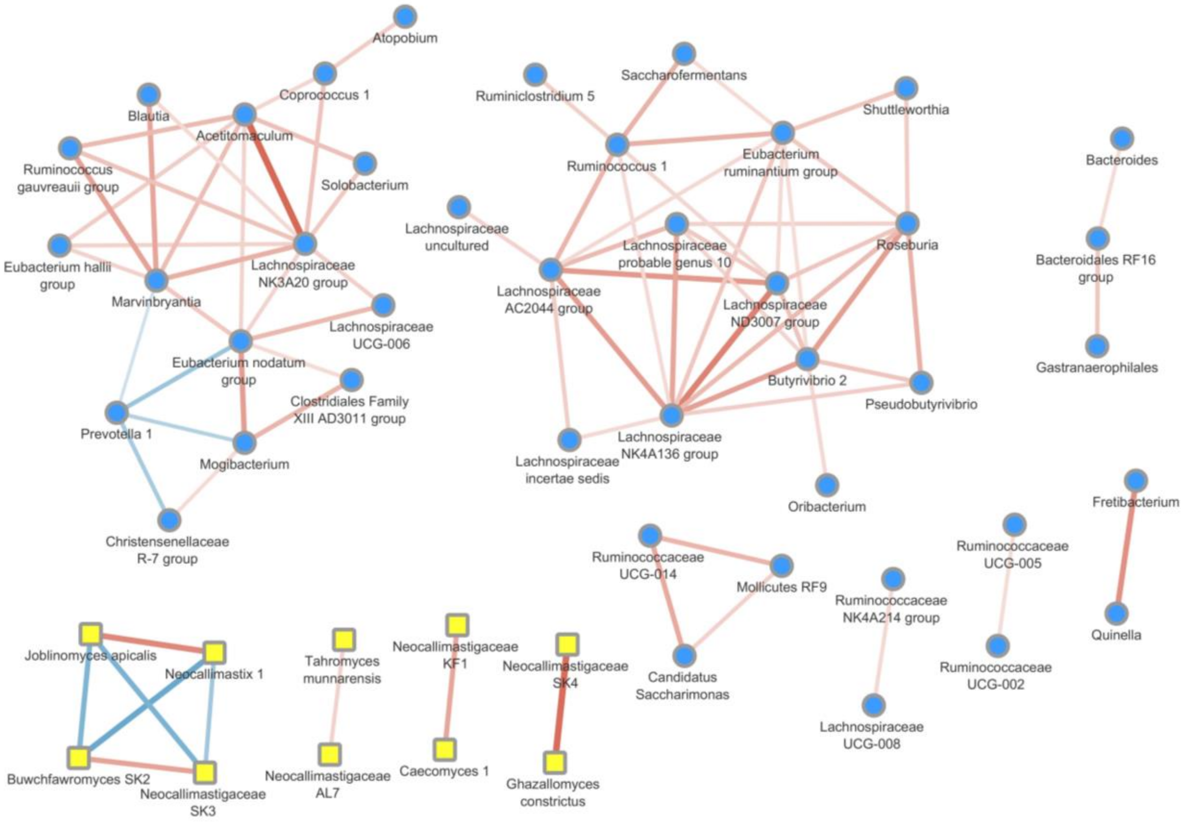

b

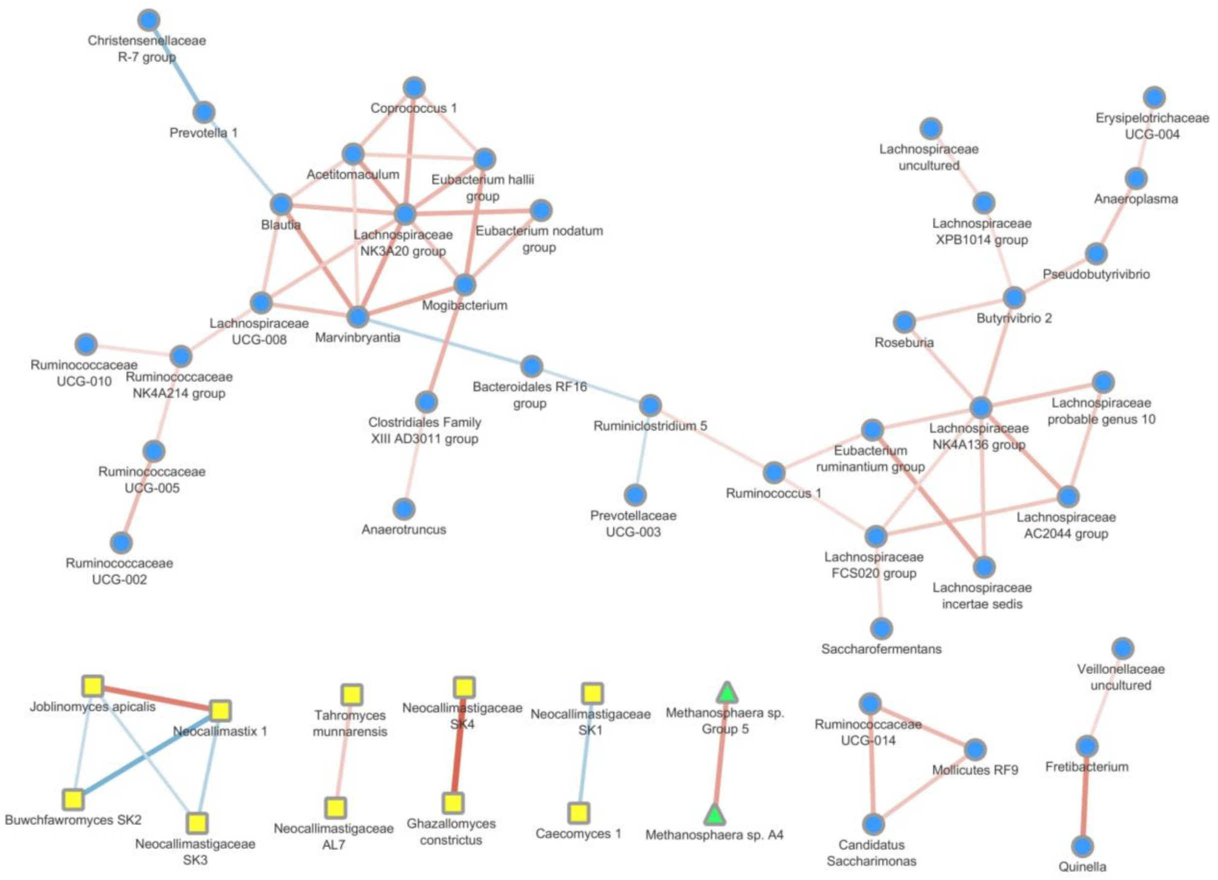
Christensenellaceae R-7 group
Prevotella 1
Coprococcus 1
Acetitomaculum
Eubacterium hallii group
Blautia
Lachnospiraceae NK3A20 group
Eubacterium nodatum group
Mogibacterium
Lachnospiraceae UCG-008
Marvinbryantia
Ruminococcaceae UCG-010
Ruminococcaceae NK4A214 group
Bacteroidales RF16 group
Clostridiales Family XIII AD3011 group
Ruminiclostridium 5
Ruminococcaceae UCG-005
Ruminococcaceae UCG-002
Anaerotruncus
Prevotellaceae UCG-003
Lachnospiraceae uncultured
Erysipelotrichaceae UCG-004
Anaeroplasma
Lachnospiraceae XPB1014 group
Pseudobutyrivibrio
Roseburia
Butyrivibrio 2
Lachnospiraceae probable genus 10
Lachnospiraceae NK4A136 group
Eubacterium ruminantium group
Ruminococcus 1
Lachnospiraceae AC2044 group
Lachnospiraceae FCS020 group
Lachnospiraceae incertae sedis
Saccharofermentans
Joblinomyces apicalis
Neocallimastix 1
Buwchfawromyces SK2
Neocallimastigaceae SK3
Tahromyces munnarensis
Neocallimastigaceae AL7
Neocallimastigaceae SK4
Ghazallomyces constrictus
Neocallimastigaceae SK1
Caecomyces 1
Methanosphaera sp. Group 5
Methanosphaera sp. A4
Ruminococcaceae UCG-014
Mollicutes RF9
Candidatus Saccharimonas
Veillonellaceae uncultured
Fretibacterium
Quinella

c

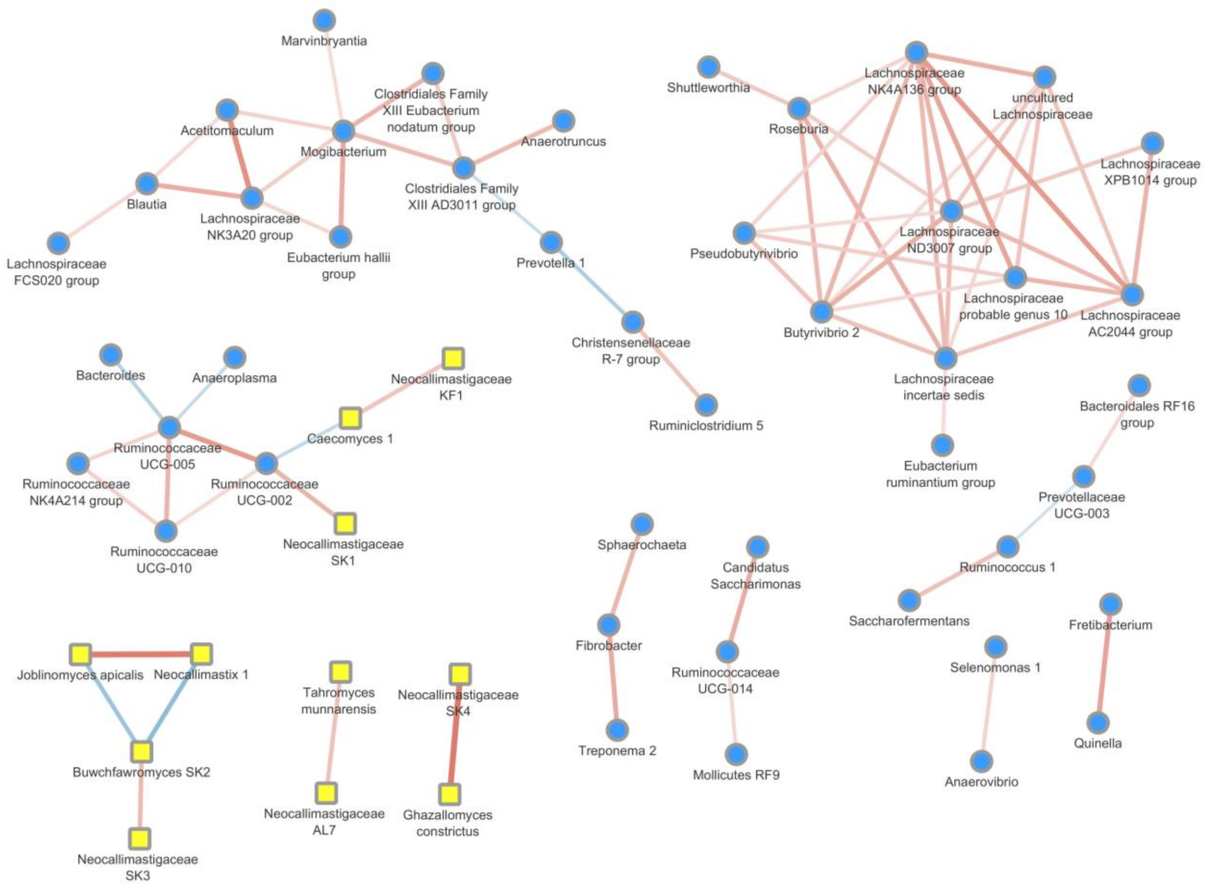

Network diagrams of Spearman correlations between microbial taxa within treatment groups (**a**) CON, (**b**) CRC and (**c**) LAI, at days 35 and 77. Graphs show correlation (r) cut offs of > 0.6 and $P < 0.05$. Blue circles, green triangles and yellow squares represent bacteria, archaea and fungi, respectively. Red and blue edges represent positive and negative correlations between adjoining nodes, respectively. Line colour intensity increases with the absolute value of r.

Supplementary Table S1. Sequence read abundances for ewe rumen microbiota.

Microbial group	Total reads[a]	Reads per sample[a] (mean ± SD[b])	Total reads from selected ewes[c]	Reads per sample from selected ewes[c] (mean ± SD[b])
Bacteria	1,328,3424	44,214 ± 25,005	10,305,904	40,735 ± 22,374
Archaea	3,383,035	11,220 ± 6,472	2,038,328	8,057 ± 5,032
Protozoa	3,571,271	11,871 ± 5,541	1,937,945	7,660 ± 3,530
Fungi			19,368,515	59,595 ± 31,058

[a]Read numbers for the classified rumen microbiota data for all 300 ewes on day 0.

[b]Standard deviation.

[c]Read numbers for the rumen microbiota of the 108 selected ewes for analysis of the bacteria, archaea and protozoa on days 35 and 77; and for fungi on days 0, 35 and 77.

Supplementary Table S2. Treatment groups means and REML analyses for ewes that were selected for microbial community analysis at D0, D35 and D77.

	Liveweight	Body condition score
Mean ± SEM[a], D77		
LAI	75.59 ± 0.65 kg	4.832 ± 0.074
CRC	75.77 ± 0.65 kg	4.862 ± 0.074
CON	74.37 ± 0.65 kg	4.791 ± 0.074
Significance (P-value[b])		
Treatment	0.1727	0.3917
Time	<.0001***	<.0001***
Co-variates (P-value[b])		
Liveweight (D0)	<.0001***	0.8158
Body condition score (D0)	0.9143	0.0004**
Ewe age	0.4260	0.8914
Treatment x time	0.9713	0.1631
Treatment x liveweight (D0)	0.0041**	0.8153
Treatment x body condition score (D0)	0.0660	0.4988
Treatment x ewe age	0.2697	0.5789

[a]Standard error of the mean

[b]*P*-values from REML models are shown ($P < 0.05$, *; $P < 0.01$, **; $P < 0.001$, ***).

Supplementary Table S3. ANOVA and least significant difference analysis of relative microbial taxon abundance (as percentage) at day 35.

Taxon[a]	CON		CRC			LAI			*P*-value[c]	FDR
	Mean	SEM	Mean	SEM	FC[b]	Mean	SEM	FC[b]		
Bacteria										
Clostridiales Family XIII AD3011 group	0.32[a]	0.014	0.27[b]	0.012	0.85	0.26[b]	0.011	0.82	0.002**	0.045
Papillibacter	0.13[b]	0.008	0.17[a]	0.009	1.27	0.13[b]	0.012	0.97	0.005**	0.085
Bacteria candidate division SR1	0.24[b]	0.016	0.34[a]	0.026	1.42	0.30[ab]	0.022	1.25	0.006**	0.085
Pseudobutyrivibrio	1.41[a]	0.074	1.16[b]	0.069	0.82	1.14[b]	0.058	0.81	0.010*	0.114
Ruminococcaceae UCG-005	1.12[a]	0.052	1.11[a]	0.045	0.99	0.94[b]	0.041	0.84	0.011*	0.114
Ruminococcaceae uncultured	0.10[b]	0.005	0.12[a]	0.005	1.19	0.11[ab]	0.005	1.07	0.021*	0.191
Butyrivibrio 2	1.02[a]	0.048	0.86[b]	0.039	0.84	0.89[b]	0.043	0.87	0.023*	0.193
Shuttleworthia	0.13[a]	0.007	0.11[b]	0.009	0.82	0.11[b]	0.005	0.81	0.025*	0.195
Atopobium	0.37[a]	0.053	0.33[a]	0.047	0.91	0.21[b]	0.022	0.59	0.029*	0.218
Lachnospiraceae probable genus 10	0.39	0.024	0.34	0.017	0.86	0.35	0.018	0.88	0.107	0.564
Ruminococcaceae UCG-002	0.54	0.021	0.59	0.026	1.08	0.52	0.020	0.96	0.108	0.564

Bacteroidales S24-7 group	2.56	0.138	2.42	0.104	0.95	2.21	0.112	0.86	0.119	0.564
Clostridiales Family XIII *Mogibacterium*	0.41	0.022	0.38	0.018	0.92	0.36	0.018	0.86	0.121	0.564
Bacteroides	0.36	0.014	0.41	0.019	1.14	0.38	0.022	1.05	0.135	0.602
Defluviitaleaceae UCG-011	0.10	0.007	0.10	0.006	1.04	0.12	0.008	1.17	0.140	0.602
Treponema 2	0.54	0.026	0.58	0.029	1.09	0.50	0.032	0.94	0.148	0.602
Bacteroidales BS11 gut group	3.74	0.219	3.95	0.185	1.06	4.47	0.382	1.19	0.163	0.617
Erysipelotrichaceae UCG-004	0.20	0.013	0.24	0.015	1.17	0.24	0.016	1.16	0.174	0.617
Fretibacterium	0.23	0.034	0.34	0.048	1.43	0.33	0.050	1.42	0.184	0.617
Oligoflexales	0.15	0.023	0.17	0.033	1.14	0.10	0.019	0.71	0.187	0.617
Coprococcus 1	0.12	0.007	0.12	0.008	0.97	0.10	0.006	0.86	0.190	0.617
Clostridiales Family XIII *Anaerovorax*	0.19	0.008	0.20	0.007	1.05	0.18	0.009	0.94	0.191	0.617
Ruminococcaceae NK4A214 group	3.28	0.127	3.36	0.134	1.02	3.05	0.138	0.93	0.235	0.689
Saccharofermentans	1.03	0.051	0.93	0.044	0.90	0.94	0.046	0.91	0.245	0.701
Quinella	3.76	0.515	4.21	0.460	1.12	4.86	0.457	1.29	0.267	0.728
Eubacterium hallii group	0.13	0.007	0.13	0.007	0.99	0.11	0.006	0.89	0.272	0.728
Rikenellaceae RC9 gut group	4.41	0.126	4.19	0.096	0.95	4.23	0.089	0.96	0.299	0.746

Prevotellaceae NK3B31 group	0.46	0.034	0.45	0.036	0.98	0.56	0.087	1.23	0.300	0.746
Prevotellaceae uncultured	0.10	0.009	0.13	0.015	1.23	0.13	0.014	1.23	0.307	0.746
Acetitomaculum	0.23	0.014	0.25	0.019	1.09	0.22	0.016	0.94	0.334	0.795
Ruminococcaceae UCG-014	1.04	0.062	1.15	0.057	1.10	1.05	0.063	1.01	0.385	0.869
Marvinbryantia	0.12	0.007	0.11	0.007	0.95	0.11	0.006	0.90	0.408	0.869
Lachnospiraceae NK3A20 group	0.90	0.046	0.81	0.037	0.91	0.85	0.049	0.95	0.423	0.869
Veillonellaceae uncultured	0.11	0.009	0.12	0.007	1.10	0.12	0.007	1.12	0.455	0.869
Lachnospiraceae NK4A136 group	0.52	0.023	0.49	0.020	0.94	0.48	0.023	0.93	0.458	0.869
Prevotellaceae UCG-004	0.14	0.016	0.14	0.014	0.97	0.12	0.009	0.85	0.461	0.869
Eubacterium ruminantium group	0.87	0.042	0.81	0.031	0.93	0.82	0.031	0.95	0.470	0.869
Ruminiclostridium 5	0.50	0.022	0.50	0.019	1.00	0.47	0.018	0.94	0.471	0.869
Thalassospira	0.46	0.028	0.49	0.036	1.07	0.44	0.024	0.96	0.475	0.869
Ruminococcus 2	0.30	0.024	0.27	0.018	0.91	0.27	0.016	0.90	0.477	0.869
Roseburia	0.58	0.023	0.56	0.022	0.97	0.54	0.022	0.94	0.496	0.869
Family Candidatus *Saccharimonas*	0.52	0.034	0.53	0.021	1.02	0.48	0.022	0.94	0.496	0.869
Eubacterium coprostanoligenes group	1.25	0.047	1.23	0.039	0.98	1.30	0.044	1.04	0.519	0.869

Anaerotruncus	0.13	0.006	0.13	0.006	0.97	0.12	0.005	0.93	0.520	0.869
Gastranaerophilales	0.96	0.058	0.88	0.039	0.92	0.90	0.048	0.94	0.532	0.869
Ruminiclostridium 9	0.11	0.008	0.11	0.007	1.01	0.10	0.007	0.91	0.542	0.869
Ruminiclostridium 6	0.14	0.008	0.14	0.011	0.98	0.13	0.007	0.91	0.546	0.869
Ruminococcaceae UCG-010	0.67	0.022	0.66	0.024	0.98	0.64	0.025	0.95	0.553	0.869
Bacteria SHA 109	0.09	0.008	0.10	0.008	1.10	0.09	0.007	1.00	0.568	0.869
Prevotellaceae UCG-001	2.03	0.094	1.94	0.110	0.96	1.89	0.072	0.93	0.578	0.869
Succinivibrionaceae uncultured	0.11	0.007	0.11	0.006	1.01	0.12	0.007	1.08	0.581	0.869
Chloroplast	0.11	0.028	0.15	0.035	1.41	0.12	0.030	1.15	0.586	0.869
Prevotellaceae UCG-003	1.38	0.092	1.31	0.048	0.95	1.29	0.058	0.93	0.615	0.878
Oribacterium	0.40	0.019	0.39	0.025	0.99	0.37	0.018	0.93	0.624	0.878
Lachnospiraceae AC2044 group	0.75	0.041	0.73	0.034	0.97	0.70	0.036	0.93	0.638	0.878
Christensenellaceae R-7 group	13.17	0.451	12.50	0.594	0.95	12.88	0.451	0.98	0.639	0.878
Prevotella 1	23.73	0.848	24.84	0.827	1.05	24.55	0.906	1.03	0.640	0.878
Oscillospira	0.19	0.034	0.24	0.057	1.24	0.25	0.053	1.32	0.641	0.878
Anaerovibrio	0.08	0.007	0.08	0.004	1.02	0.08	0.005	0.94	0.663	0.880

Bacteroidales UCG-001	0.39	0.031	0.36	0.025	0.92	0.36	0.026	0.93	0.693	0.884
Erysipelotrichaceae UCG-009	0.10	0.006	0.10	0.007	0.97	0.09	0.007	0.93	0.706	0.884
Blautia	0.16	0.008	0.15	0.007	0.94	0.15	0.010	0.99	0.711	0.884
Mollicutes RF9	1.25	0.054	1.30	0.048	1.04	1.27	0.043	1.01	0.717	0.884
Clostridiales Family XIII *Eubacterium nodatum* group	0.12	0.008	0.11	0.005	0.96	0.11	0.005	0.95	0.724	0.884
Sphaerochaeta	0.21	0.023	0.19	0.017	0.90	0.20	0.023	0.95	0.744	0.884
Lachnospiraceae FCS020 group	0.15	0.012	0.16	0.008	1.01	0.15	0.010	0.95	0.749	0.884
Ruminococcus 1	3.00	0.140	2.88	0.083	0.96	2.91	0.137	0.97	0.749	0.884
Lachnospiraceae UCG-008	0.19	0.007	0.19	0.010	0.96	0.19	0.009	0.97	0.751	0.884
Anaerolineaceae uncultured	0.13	0.007	0.13	0.007	0.98	0.14	0.007	1.03	0.751	0.884
Ruminococcus gauvreauii group	0.37	0.018	0.37	0.016	0.98	0.38	0.017	1.02	0.787	0.896
Lachnospiraceae incertae sedis	0.17	0.010	0.16	0.008	0.95	0.16	0.009	0.97	0.792	0.896
Lachnospiraceae XPB1014 group	0.72	0.048	0.67	0.044	0.93	0.69	0.061	0.96	0.795	0.896
Lachnospiraceae uncultured	0.49	0.016	0.49	0.013	0.99	0.50	0.018	1.02	0.803	0.896
Selenomonas 1	0.96	0.056	0.95	0.054	0.99	1.00	0.057	1.04	0.804	0.896

Fibrobacter	2.37	0.144	2.33	0.125	0.98	2.45	0.197	1.03	0.855	0.924
Lachnospiraceae UCG-006	0.16	0.038	0.14	0.011	0.88	0.16	0.035	0.96	0.891	0.924
Anaeroplasma	0.29	0.018	0.28	0.020	0.96	0.29	0.020	0.99	0.892	0.924
Victivallis	0.25	0.017	0.24	0.016	0.97	0.24	0.016	0.96	0.902	0.924
Lachnospiraceae ND3007 group	0.49	0.022	0.50	0.024	1.03	0.49	0.026	1.00	0.902	0.924
Bacteroidales RF16 group	1.99	0.237	1.98	0.178	0.99	1.89	0.132	0.95	0.918	0.933
Solobacterium	0.19	0.015	0.19	0.010	1.02	0.19	0.015	1.01	0.978	0.986
Succiniclasticum	1.09	0.036	1.08	0.035	1.00	1.09	0.042	1.01	0.988	0.988
Bacteria minor	5.42	0.116	5.36	0.125	0.99	5.19	0.120	0.96	0.399	0.869
Archaea										
Methanomassiliicoccaceae Group 12	1.32[b]	0.104	4.72[a]	0.320	3.57	1.07[b]	0.124	0.81	<0.001[**]	0.000
Methanosphaera sp. Group 5	2.06[a]	0.209	0.28[b]	0.038	0.14	1.94[a]	0.174	0.94	<0.001[**]	0.000
Methanomassiliicoccaceae Group 9	3.08[a]	0.260	1.56[b]	0.120	0.51	3.03[a]	0.236	0.98	<0.001[**]	0.000
Methanosphaera sp. ISO-3F5	3.41[b]	0.303	5.17[a]	0.308	1.51	3.03[b]	0.235	0.89	<0.001[**]	0.000
Methanobrevibacter gottschalkii clade	49.66[a]	1.029	44.55[b]	0.892	0.90	51.21[a]	1.110	1.03	<0.001[**]	0.001
Methanobrevibacter ruminantium clade	19.73[b]	0.909	24.69[a]	1.022	1.25	19.60[b]	0.972	0.99	<0.001[**]	0.005

Methanomassiliicoccaceae Group 10	13.95[a]	0.627	11.80[b]	0.450	0.85	13.13[ab]	0.530	0.94	0.020[*]	0.191
Methanomassiliicoccaceae Group 4	3.98[b]	0.229	4.75[a]	0.209	1.19	4.12[b]	0.225	1.04	0.036[*]	0.255
Methanomassiliicoccaceae Group 11	0.65	0.067	0.52	0.047	0.80	0.68	0.068	1.05	0.145	0.602
Methanosphaera sp. A4	1.25	0.139	1.08	0.138	0.86	1.30	0.133	1.04	0.492	0.869
Methanomassiliicoccaceae Group 8	0.72	0.058	0.68	0.054	0.94	0.69	0.069	0.95	0.876	0.924
Archaea minor	0.13	0.016	0.16	0.024	1.15	0.14	0.010	1.02	0.659	0.880
Protozoa										
Diploplastron/Eremoplastron	0.05[a]	0.006	0.03[b]	0.005	0.63	0.06[a]	0.008	1.20	0.007[**]	0.085
Metadinium	0.01	0.003	0.01	0.002	0.67	0.01	0.003	0.64	0.204	0.641
Anoplodinium/Diplodinium	14.44	2.138	20.69	2.606	1.43	17.92	2.682	1.24	0.209	0.644
Enoploplastron	0.09	0.015	0.12	0.021	1.23	0.13	0.022	1.37	0.414	0.869
Eudiplodinium	24.74	1.931	22.17	1.941	0.90	21.2	1.964	0.86	0.415	0.869
Isotricha	0.11	0.038	0.09	0.046	0.86	0.05	0.018	0.46	0.456	0.869
Epidinium	39.41	1.742	36.18	2.430	0.92	38.53	2.145	0.98	0.542	0.869
Polyplastron	0.02	0.006	0.01	0.004	0.72	0.01	0.004	0.78	0.581	0.869
Entodinium	16.70	0.995	16.63	1.045	1.00	17.42	0.977	1.04	0.826	0.913

Dasytricha	4.35	0.894	4.00	0.510	0.92	4.59	0.833	1.05	0.858	0.924
Protozoa minor	0.02	0.004	0.02	0.005	1.18	0.02	0.003	1.18	0.677	0.884
Fungi										
Neocallimastigaceae KF1	0.35	0.08	0.12	0.04	0.35	0.23	0.07	0.66	0.07[†]	0.444
Buwchfawromyces SK2	11.17	1.49	17.29	2.47	1.55	14.67	1.80	1.31	0.09[†]	0.578
Neocallimastigaceae AL7	0.13	0.01	0.14	0.01	1.15	0.14	0.01	1.08	0.14	0.602
Neocallimastix 1	14.05	2.22	10.86	2.11	0.77	8.64	1.35	0.62	0.14	0.602
Neocallimastigaceae SK4	18.01	2.13	12.88	1.50	0.71	15.77	1.91	0.88	0.15	0.605
Joblinomyces apicalis	0.13	0.02	0.10	0.01	0.76	0.10	0.01	0.82	0.23	0.689
Piromyces 1	1.92	0.47	3.03	0.63	1.57	2.96	0.40	1.54	0.23	0.689
Neocallimastigaceae JF423626	0.01	0.00	0.43	0.24	38.97	1.07	0.79	98.4	0.29	0.766
Neocallimastigaceae SK3	13.36	1.02	14.24	0.99	1.07	15.38	0.92	1.15	0.35	0.843
Neocallimastigaceae BlackRhino	0.23	0.06	0.16	0.05	0.70	0.13	0.03	0.6	0.41	0.863
Ghazallomyces constrictus	0.20	0.02	0.16	0.02	0.82	0.19	0.02	0.96	0.43	0.863
Caecomyces 1	10.63	1.28	10.96	1.36	1.03	12.51	1.21	1.18	0.55	0.863
Tahromyces munnarensis	1.81	0.08	1.92	0.09	1.06	1.85	0.07	1.02	0.59	0.863

Feramyces austinii	0.03	0.01	0.09	0.04	3.00	0.08	0.07	2.53	0.61	0.869
Piromyces 7	6.58	0.84	6.11	0.74	0.93	5.76	0.59	0.87	0.72	0.876
Piromyces 2	16.29	1.72	16.09	1.33	0.99	15.29	1.21	0.94	0.87	0.931
Neocallimastigaceae SK1	4.17	0.34	4.39	0.40	1.05	4.32	0.37	1.03	0.91	0.933
Fungi minor	0.23	0.01	0.25	0.05	1.06	0.19	0.01	0.84	0.49	0.863

[a]Data are the means and standard errors of the means (SEM) of the relative abundances of each microbial taxon.

[b]Fold changes (FC) of mean CRC to CON and LAI to CON relative abundances are shown.

[c]P-values from one-way ANOVA test are shown ($P < 0.01$, **; $P < 0.05$, *; $P < 0.1$, †). Least significant difference *post-hoc* tests were conducted on taxa with ANOVA $P < 0.05$. Different letters (a, b) indicate significant differences between treatments for taxon.

Supplementary Table S4. ANOVA and least significant difference analysis of relative microbial taxon abundance (as percentage) at day 77.

Taxon[a]	CON		CRC			LAI			*P*-value[c]	FDR
	Mean	SEM	Mean	SEM	FC[b]	Mean	SEM	FC[b]		
Bacteria										
Papillibacter	0.151[b]	0.0068	0.196[a]	0.0077	1.29	0.129[c]	0.0074	0.85	<0.001**	0.000
Anaerolineaceae uncultured	0.169[a]	0.0068	0.168[a]	0.0085	0.99	0.137[b]	0.0070	0.81	0.004**	0.044
Mollicutes RF9	1.263[a]	0.0580	1.066[b]	0.0447	0.84	1.285[a]	0.0456	1.02	0.004**	0.044
Selenomonas 1	1.192[b]	0.0684	1.298[b]	0.0719	1.09	1.545[a]	0.0874	1.30	0.005**	0.050
Family Candidatus *Saccharimonas*	0.563[a]	0.0250	0.466[b]	0.0233	0.83	0.555[a]	0.0242	0.99	0.009**	0.092
Bacteroides	0.272[ab]	0.0120	0.298[a]	0.0143	1.10	0.245[b]	0.0119	0.90	0.015*	0.121
Pseudobutyrivibrio	0.907[a]	0.0622	0.699[b]	0.0429	0.77	0.763[ab]	0.0476	0.84	0.016*	0.121
Clostridiales Family XIII *Mogibacterium*	0.548[a]	0.0244	0.482[b]	0.0212	0.88	0.471[b]	0.0177	0.86	0.023*	0.139
Marvinbryantia	0.145[ab]	0.0072	0.152[a]	0.0068	1.05	0.126[b]	0.0063	0.87	0.025*	0.142
Veillonellaceae uncultured	0.132[b]	0.0093	0.130[b]	0.0097	0.99	0.170[a]	0.0149	1.29	0.027*	0.142
Atopobium	0.434[a]	0.0346	0.445[a]	0.0385	1.03	0.318[b]	0.0440	0.73	0.045*	0.211

Anaerotruncus	0.160[a]	0.0067	0.140[b]	0.0060	0.88	0.143[b]	0.0055	0.89	0.047[*]	0.213
Fibrobacter	2.410	0.1273	2.209	0.1226	0.92	1.969	0.1291	0.82	0.051[†]	0.223
Succiniclasticum	1.137	0.0432	1.128	0.0377	0.99	1.244	0.0338	1.09	0.064[†]	0.261
Coprococcus 1	0.150	0.0086	0.145	0.0081	0.97	0.125	0.0069	0.84	0.072[†]	0.267
Butyrivibrio 2	0.893	0.0424	0.765	0.0354	0.86	0.820	0.0397	0.92	0.074[†]	0.267
Rikenellaceae RC9 gut group	4.534	0.1682	4.295	0.1724	0.95	4.021	0.1309	0.89	0.076[†]	0.267
Lachnospiraceae uncultured	0.490	0.0154	0.442	0.0132	0.90	0.476	0.0169	0.97	0.080[†]	0.267
Lachnospiraceae XPB1014 group	0.716	0.0536	0.6	0.0362	0.85	0.599	0.0262	0.84	0.081[†]	0.267
Shuttleworthia	0.120	0.0065	0.10	0.0048	0.88	0.126	0.0082	1.06	0.083[†]	0.267
Quinella	3.858	0.4727	4.31	0.5036	1.12	5.325	0.4804	1.38	0.096[†]	0.304
Bacteria SHA-109	0.162	0.0111	0.133	0.0081	0.82	0.147	0.0093	0.91	0.115	0.339
Anaerovibrio	0.082	0.0050	0.094	0.0057	1.14	0.100	0.0074	1.21	0.134	0.369
Prevotellaceae UCG-004	0.152	0.0147	0.162	0.0151	1.06	0.125	0.0088	0.83	0.135	0.369
Saccharofermentans	0.912	0.0341	0.822	0.0309	0.90	0.882	0.0322	0.97	0.141	0.373
Prevotellaceae UCG-001	1.921	0.0855	1.984	0.0780	1.03	1.766	0.0737	0.92	0.142	0.373
Acetitomaculum	0.284	0.0161	0.289	0.0158	1.02	0.251	0.0124	0.88	0.147	0.377

Eubacterium hallii group	0.154	0.0086	0.165	0.0087	1.07	0.143	0.0064	0.93	0.155	0.391
Oribacterium	0.448	0.0265	0.392	0.0207	0.87	0.418	0.0164	0.93	0.190	0.451
Blautia	0.177	0.0106	0.188	0.0098	1.06	0.163	0.0083	0.92	0.194	0.451
Anaeroplasma	0.239	0.0157	0.213	0.0128	0.89	0.209	0.0099	0.87	0.212	0.451
Ruminococcaceae UCG-010	0.884	0.0213	0.829	0.0207	0.94	0.854	0.0242	0.97	0.216	0.451
Gastranaerophilales	0.883	0.0441	0.784	0.0388	0.89	0.866	0.0438	0.98	0.216	0.451
Clostridiales Family XIII AD3011 group	0.379	0.0187	0.349	0.0140	0.92	0.343	0.0135	0.90	0.217	0.451
Prevotella 1	21.324	0.7571	23.021	0.6415	1.08	22.803	0.8187	1.07	0.218	0.451
Eubacterium coprostanoligenes group	1.407	0.0406	1.337	0.0403	0.95	1.430	0.0386	1.02	0.230	0.468
Victivallis	0.264	0.0149	0.230	0.0178	0.87	0.227	0.0177	0.86	0.242	0.483
Ruminococcaceae UCG-014	1.059	0.0536	0.955	0.0512	0.90	1.053	0.0429	0.99	0.250	0.493
Lachnospiraceae UCG-008	0.226	0.0099	0.208	0.0069	0.92	0.211	0.0088	0.93	0.283	0.541
Lachnospiraceae FCS020 group	0.152	0.0094	0.132	0.0069	0.87	0.142	0.0103	0.93	0.299	0.554
Bacteroidales S24-7 group	2.992	0.1701	2.838	0.1264	0.95	3.173	0.1557	1.06	0.299	0.554
Thalassospira	0.544	0.0349	0.523	0.0304	0.96	0.477	0.0282	0.88	0.306	0.556
Ruminococcaceae UCG-005	1.501	0.0521	1.449	0.0659	0.96	1.380	0.0551	0.92	0.320	0.562

Prevotellaceae NK3B31 group	0.484	0.0311	0.533	0.0316	1.10	0.563	0.0472	1.16	0.321	0.562
Ruminococcus gauvreauii group	0.433	0.0212	0.473	0.0204	1.09	0.443	0.0176	1.02	0.339	0.579
Ruminococcaceae NK4A214 group	3.963	0.1745	3.959	0.1332	1.00	3.687	0.1461	0.93	0.344	0.579
Christensenellaceae R-7 group	13.69	0.4751	13.319	0.4867	0.97	12.720	0.4775	0.93	0.352	0.583
Fretibacterium	0.360	0.0446	0.443	0.0711	1.23	0.468	0.0467	1.30	0.360	0.589
Defluviitaleaceae UCG-011	0.101	0.0057	0.090	0.0044	0.89	0.096	0.0065	0.95	0.375	0.598
Ruminiclostridium 5	0.486	0.0172	0.505	0.0174	1.04	0.521	0.0190	1.07	0.386	0.607
Solobacterium	0.208	0.0172	0.194	0.0104	0.93	0.183	0.0103	0.88	0.403	0.624
Ruminococcaceae UCG-002	0.831	0.0348	0.857	0.0385	1.03	0.898	0.0324	1.08	0.406	0.624
Lachnospiraceae NK4A136 group	0.453	0.0156	0.423	0.0181	0.93	0.425	0.0190	0.94	0.413	0.627
Clostridiales Family XIII *Eubacterium nodatum* group	0.142	0.0061	0.146	0.0057	1.03	0.136	0.0043	0.96	0.427	0.631
Lachnospiraceae NK3A20 group	1.130	0.0606	1.091	0.0631	0.97	1.026	0.0459	0.91	0.430	0.631
Lachnospiraceae UCG-006	0.155	0.0076	0.147	0.0115	0.95	0.141	0.0055	0.91	0.492	0.690
Ruminiclostridium 9	0.170	0.0080	0.189	0.0164	1.10	0.171	0.0109	1.00	0.499	0.690
Oscillospira	0.279	0.0493	0.346	0.0276	1.24	0.316	0.0424	1.13	0.509	0.690

Treponema 2	0.433	0.0233	0.451	0.0275	1.04	0.412	0.0210	0.95	0.534	0.715
Clostridiales Family XIII *Anaerovorax*	0.192	0.0095	0.184	0.0063	0.96	0.179	0.0070	0.94	0.539	0.715
Roseburia	0.515	0.0188	0.524	0.0235	1.02	0.547	0.0223	1.06	0.552	0.724
Bacteroidales BS11 gut group	3.957	0.1357	4.033	0.2481	1.02	3.774	0.1169	0.95	0.568	0.732
Ruminococcus 1	2.673	0.0897	2.743	0.0980	1.03	2.808	0.0806	1.05	0.569	0.732
Erysipelotrichaceae UCG-009	0.138	0.0063	0.131	0.0081	0.95	0.140	0.0066	1.01	0.608	0.758
Ruminococcus 2	0.198	0.0095	0.194	0.0099	0.98	0.208	0.0105	1.05	0.615	0.760
Ruminiclostridium 6	0.146	0.0094	0.160	0.0149	1.10	0.148	0.0074	1.02	0.635	0.777
Oligoflexales	0.165	0.0233	0.134	0.0211	0.81	0.142	0.0290	0.86	0.649	0.786
Bacteroidales UCG-001	0.307	0.0187	0.313	0.0166	1.02	0.292	0.0161	0.95	0.675	0.803
Ruminococcaceae uncultured	0.137	0.0069	0.145	0.0068	1.06	0.145	0.0078	1.06	0.676	0.803
Prevotellaceae UCG-003	1.361	0.0548	1.346	0.0448	0.99	1.304	0.0522	0.96	0.716	0.825
Erysipelotrichaceae UCG-004	0.242	0.0133	0.241	0.0136	0.99	0.277	0.0590	1.14	0.721	0.825
Succinivibrionaceae uncultured	0.110	0.0061	0.114	0.0056	1.04	0.108	0.0061	0.98	0.727	0.825
Lachnospiraceae probable genus 10	0.280	0.0160	0.262	0.0158	0.94	0.272	0.0180	0.97	0.740	0.825
Chloroplast	0.167	0.0438	0.217	0.0547	1.30	0.180	0.0633	1.09	0.804	0.874

Eubacterium ruminantium group	0.750	0.0335	0.745	0.0446	0.99	0.778	0.0363	1.04	0.804	0.874
Lachnospiraceae ND3007 group	0.454	0.0175	0.441	0.0211	0.97	0.457	0.0234	1.01	0.845	0.910
Sphaerochaeta	0.190	0.0159	0.185	0.0143	0.95	0.190	0.0159	0.97	0.904	0.966
Prevotellaceae uncultured	0.132	0.0125	0.136	0.0095	1.03	0.139	0.0118	1.05	0.917	0.971
Lachnospiraceae incertae sedis	0.148	0.0098	0.145	0.0109	0.98	0.149	0.0098	1.01	0.960	0.981
Bacteroidales RF16 group	1.242	0.0983	1.260	0.0884	1.01	1.223	0.0927	0.98	0.962	0.981
Lachnospiraceae AC2044 group	0.598	0.0273	0.607	0.0368	1.02	0.608	0.0266	1.02	0.965	0.981
Bacteria candidate division SR1	0.378	0.0206	0.380	0.0295	1.01	0.377	0.0186	1.00	0.996	1.000
Bacteria minor	6.017	0.1095	5.799	0.0961	0.96	5.706	0.1162	0.95	0.116	0.339
Archaea										
Methanomassiliicoccaceae Group 12	1.471[b]	0.1460	5.223[a]	0.3346	3.55	1.356[b]	0.1115	0.92	<0.001**	0.000
Methanosphaera sp. Group 5	2.309[a]	0.1983	0.451[b]	0.0885	0.20	2.251[a]	0.1846	0.97	<0.001**	0.000
Methanobrevibacter ruminantium clade	19.368[b]	0.8416	23.500[a]	0.8334	1.21	17.138[c]	0.6295	0.88	<0.001**	0.000
Methanobrevibacter gottschalkii clade	49.462[a]	0.8029	42.534[b]	1.1191	0.86	49.703[a]	1.0265	1.00	<0.001**	0.000
Methanosphaera sp. ISO-3F5	4.257[b]	0.2125	6.161[a]	0.4334	1.45	4.222[b]	0.2065	0.99	<0.001**	0.000
Methanomassiliicoccaceae Group 4	4.095[c]	0.2359	5.638[a]	0.2327	1.38	4.829[b]	0.2327	1.18	<0.001**	0.001

Methanomassiliicoccaceae Group 9	3.278[a]	0.3306	2.048[b]	0.1610	0.62	3.444[a]	0.2601	1.05	<0.001**	0.005
Methanomassiliicoccaceae Group 10	12.765[a]	0.5992	10.978[b]	0.6002	0.86	14.021[a]	0.6111	1.10	0.002**	0.033
Methanosphaera sp. A4	1.224[b]	0.1222	1.761[a]	0.1870	1.44	1.169[b]	0.1513	0.96	0.015*	0.121
Methanomassiliicoccaceae Group 11	0.807[b]	0.0579	0.761[b]	0.0714	0.94	1.017[a]	0.0726	1.26	0.020*	0.131
Methanomassiliicoccaceae Group 8	0.809	0.0762	0.759	0.0591	0.94	0.718	0.0498	0.89	0.590	0.751
Archaea minor	0.148[ab]	0.0133	0.180[a]	0.0123	1.22	0.128[b]	0.0132	0.87	0.019*	0.131
Protozoa										
Eudiplodinium	14.59[ab]	1.3880	11.910[b]	1.3325	0.82	17.844[a]	1.6987	1.22	0.021*	0.131
Enoploplastron	0.094	0.0136	0.095	0.0141	1.01	0.136	0.0158	1.44	0.076†	0.267
Polyplastron	0.020	0.0052	0.032	0.0073	1.56	0.017	0.0042	0.87	0.173	0.427
Diploplastron/Eremoplastron	0.035	0.0044	0.046	0.0051	1.29	0.033	0.0061	0.94	0.210	0.451
Anoplodinium/Diplodinium	27.563	1.8421	29.952	2.0154	1.09	25.204	2.4040	0.91	0.283	0.541
Entodinium	10.537	0.6988	11.532	0.5394	1.09	10.660	0.7154	1.01	0.507	0.690
Metadinium	0.009	0.0020	0.010	0.0024	1.13	0.012	0.0030	1.29	0.718	0.825
Isotricha	0.144	0.0757	0.069	0.0242	0.48	0.145	0.1122	1.01	0.739	0.825
Epidinium	40.832	2.1334	40.154	1.9681	0.98	39.781	2.0745	0.97	0.935	0.981

Dasytricha	6.132	1.4543	6.165	0.9611	1.01	6.128	0.9612	1.00	1.000	1.000
Protozoa minor	0.034	0.0041	0.029	0.0041	0.84	0.033	0.0088	0.96	0.802	0.874
Fungi										
Neocallimastigaceae SK4	27.81[a]	2.78	20.64[b]	2.13	0.74	18.79[b]	1.43	0.68	0.01[*]	0.094
Piromyces 1	0.58[b]	0.24	0.33[b]	0.16	0.57	1.85[a]	0.71	3.19	0.04[*]	0.187
Joblinomyces apicalis	0.12	0.01	0.10	0.01	0.88	0.08	0.01	0.71	0.07[†]	0.267
Neocallimastigaceae KF1	0.06	0.02	0.02	0.00	0.32	0.03	0.01	0.53	0.07[†]	0.267
Piromyces 2	15.40	1.34	18.45	1.16	1.20	19.32	1.22	1.25	0.07[†]	0.267
Neocallimastigaceae AL7	0.11	0.01	0.13	0.01	1.17	0.12	0.01	1.14	0.09[†]	0.289
Neocallimastigaceae SK1	8.01	0.47	9.73	0.61	1.21	9.06	0.56	1.13	0.09[†]	0.285
Tahromyces munnarensis	1.64	0.07	1.89	0.09	1.15	1.71	0.11	1.04	0.13	0.361
Ghazallomyces constrictus	0.27	0.03	0.24	0.03	0.87	0.21	0.02	0.78	0.25	0.485
Neocallimastigaceae JF423626	0.24	0.20	0.19	0.12	0.76	1.75	1.32	7.13	0.27	0.521
Feramyces austinii	0.22	0.14	0.47	0.27	2.17	0.09	0.08	0.41	0.32	0.547
Buwchfawromyces SK2	13.73	1.79	16.71	1.70	1.22	14.48	1.51	1.06	0.43	0.624
Neocallimastigaceae SK3	10.45	0.80	10.74	0.57	1.03	11.68	0.81	1.12	0.47	0.667

Caecomyces 1	2.42	0.37	2.40	0.29	0.99	2.89	0.33	1.20	0.50	0.687
Neocallimastigaceae BlackRhino	0.91	0.16	0.62	0.16	0.69	0.78	0.20	0.86	0.51	0.687
Neocallimastix 1	9.99	1.56	8.84	1.59	0.88	8.90	1.54	0.89	0.84	0.910
Piromyces 7	7.17	0.92	7.46	0.73	1.04	7.41	0.61	1.03	0.96	0.981
Fungi minor	0.25	0.03	0.30	0.06	1.20	0.20	0.01	0.78	0.16	0.389

[a]Data are the means and standard errors of the mean (SEM) of the relative abundances of each microbial taxon.

[b]Fold changes (FC) of mean CRC to CON and LAI to CON relative abundances are shown.

[c]*P*-values from one-way ANOVA test are shown (*P* < 0.01, **; *P* < 0.05, *; *P* < 0.1, †). Least significant difference *post-hoc* tests were conducted on taxa with ANOVA *P* < 0.05. Different letters (a, b, c) indicate significant differences between treatments for taxon.

Supplementary Table S5. Primers used in this study.

Primer name	Target group	Sequence (5'-3')	Adapter[a]	Target gene (approximate position)	Source references
Illumina_Ba9F	Bacteria	GAGTTTGATCMTGGCTCAG	A	16S rRNA gene (9 – 27[b])	Weisburg et al.[2]
Illumina_Ba515Rmod1	Bacteria	CCGCGGCKGCTGGCAC	B	16S rRNA gene (530 – 515[b])	Modified from Lane[3] by Kittelmann et al.[4]
Illumina_Ar915aF	Archaea	AGGAATTGGCGGGGGAGCAC	B	16S rRNA gene (915 – 934[b])	Casamayor et al.[5]
Illumina_Ar1386R	Archaea	GCGGTGTGTGCAAGGAGC	A	16S rRNA gene (1402 – 1385[b])	Skillman et al.[6]
Illumina_RP841F	Protozoa	GACTAGGGATTGGARTGG	B	18S rRNA gene (1035 – 1052[c])	Kittelmann et al.[7]
Illumina_Reg1302R	Protozoa	AATTGCAAAGATCTATCCC	A	18S rRNA gene (1558 – 1540[c])	Rhind et al.[8]

Illumina_MN100F2	Anaerobic fungi	TCCTACCCTTTGTGAATT	B	ITS1 (39-56[d])	Modified from Tuckwell et al.[9], this study[5]
Illumina_MNGMR2	Anaerobic fungi	CTGCGTTCTTCATCGTTGCG	A	ITS1 (40 – 21 of 5.8S rRNA gene[e])	MNGM2 from Tuckwell et al.[9]

[a]Primers had Illumina adapter A (AATGATACGGCGACCACCGAGATCTACAC) or adapter B (CAAGCAGAAGACGGCATACGAGAT) attached to the 5' end.

[b]Nucleotide positions based on the numbering of Lane[3].

[c]Nucleotide positions based on 18S rRNA sequence of *Saccharomyces cerevisiae* strain NCYC 505 (GenBank accession Z75578[10]).

[d]Nucleotide positions based on ITS1 and 5.8S rRNA gene sequence of *Neocallimastix frontalis* strain SR4 (GenBank accession AY429664[11]).

[e]Modified from Tuckwell et al.[9] by removing the two 3’-end nucleotides to improve ITS1 amplification from all known anaerobic fungal genera to date.

References

1 Williams, A. G. in *The Rumen Protozoa* (eds Alan G. Williams & Geoffrey S. Coleman) 300-316 (Springer-Verlag, 1991).

2 Weisburg, W. G., Barns, S. M., Pelletier, D. A. & Lane, D. J. 16S ribosomal DNA amplification for phylogenetic study. *J. Bacterio.l* **173**, 697-703 (1991).

3 Lane, D. J. in *Nucleic Acid Techniques in Bacterial Systematics* (eds E. Stackebrandt & M. Goodfellow) 115-175 (John Wiley and Sons, 1991).

4 Kittelmann, S. *et al.* Simultaneous amplicon sequencing to explore co-occurrence patterns of bacterial, archaeal and eukaryotic microorganisms in rumen microbial communities. *PLoS ONE* **8**, e47879, doi:10.1371/journal.pone.0047879 (2013).

5 Casamayor, E. O. *et al.* Changes in archaeal, bacterial and eukaryal assemblages along a salinity gradient by comparison of genetic fingerprinting methods in a multipond solar saltern. *Environ. Microbiol.* **4**, 338-348 (2002).

6 Skillman, L. C. *et al.* 16S ribosomal DNA-directed PCR primers for ruminal methanogens and identification of methanogens colonising young lambs. *Anaerobe* **10**, 277-285 (2004).

7 Kittelmann, S., Kirk, M. R., Jonker, A., McCulloch, A. & Janssen, P. H. Buccal swabbing as a noninvasive method to determine bacterial, archaeal, and eukaryotic microbial community structures in the rumen. *Appl. Environ. Microbiol.* **81**, 7470-7483 (2015).

8 Rhind, S. M., Archer, Z. A. & Adam, C. L. Seasonality of food intake in ruminants: recent developments in understanding. *Nutr. Res. Rev.* **15**, 43-65 (2002).

9 Tuckwell, D. S., Nicholson, M. J., McSweeney, C. S., Theodorou, M. K. & Brookman, J. L. The rapid assignment of ruminal fungi to presumptive genera using ITS1 and ITS2 RNA secondary structures to produce group-specific fingerprints. *Microbiology* **151**, 1557-1567 (2005).

10 James, S. A., Cai, J., Roberts, I. N. & Collins, M. D. A phylogenetic analysis of the genus *Saccharomyces* based on 18S rRNA gene sequences: description of *Saccharomyces*

kunashirensis sp. nov. and *Saccharomyces martiniae* sp. nov. *Int. J. Syst. Bacteriol.* **47**, 453-460 (1997).

11 Fliegerova, K., Hodrova, B. & Voigt, K. Classical and molecular approaches as a powerful tool for the characterization of rumen polycentric fungi. *Folia Microbiol. (Praha)* **49**, 157-164 (2004).
